# Enumerating metabolic pathways for the production of heterologous target chemicals in chassis organisms

**DOI:** 10.1186/1752-0509-6-10

**Published:** 2012-02-06

**Authors:** Pablo Carbonell, Davide Fichera, Shashi B Pandit, Jean-Loup Faulon

**Affiliations:** 1 iSSB, Institute of Systems and Synthetic Biology, University of Evry, Genopole Campus 1, Genavenir 6, 5 rue Henri Desbruères, 91030 EVRY Cedex, France

## Abstract

**Background:**

We consider the possibility of engineering metabolic pathways in a chassis organism in order to synthesize novel target compounds that are heterologous to the chassis. For this purpose, we model metabolic networks through hypergraphs where reactions are represented by hyperarcs. Each hyperarc represents an enzyme-catalyzed reaction that transforms set of substrates compounds into product compounds. We follow a retrosynthetic approach in order to search in the metabolic space (hypergraphs) for pathways (hyperpaths) linking the target compounds to a source set of compounds.

**Results:**

To select the best pathways to engineer, we have developed an objective function that computes the cost of inserting a heterologous pathway in a given chassis organism. In order to find minimum-cost pathways, we propose in this paper two methods based on steady state analysis and network topology that are to the best of our knowledge, the first to enumerate all possible heterologous pathways linking a target compounds to a source set of compounds. In the context of metabolic engineering, the source set is composed of all naturally produced chassis compounds (endogenuous chassis metabolites) and the target set can be any compound of the chemical space. We also provide an algorithm for identifying precursors which can be supplied to the growth media in order to increase the number of ways to synthesize specific target compounds.

**Conclusions:**

We find the topological approach to be faster by several orders of magnitude than the steady state approach. Yet both methods are generally scalable in time with the number of pathways in the metabolic network. Therefore this work provides a powerful tool for pathway enumeration with direct application to biosynthetic pathway design.

## Background

Metabolism is the process of synthesis and degradation of molecules occurring in living organisms. Metabolism is generally represented as a network where metabolites are interconnected by reactions. In order to give a functional description of metabolism, metabolic networks are often decomposed into separated parts, called metabolic pathways. The description of metabolism through metabolic pathways is useful, even though any division in pathways is arbitrary, because it helps in modeling and understanding the behavior of the full network. A metabolic pathway can be defined as a coherent set of enzyme-catalyzed biochemical reactions by which a living organism transforms a set of source compounds into a set of target compounds. By regulating enzyme and protein synthesis, living organisms can adapt to different environments. This model of metabolism as composed by independent metabolic pathways is simplistic, since pathways are nested and interdependent. In fact, metabolism is a complex system and pathways interact with each other.

**Figure 1:**
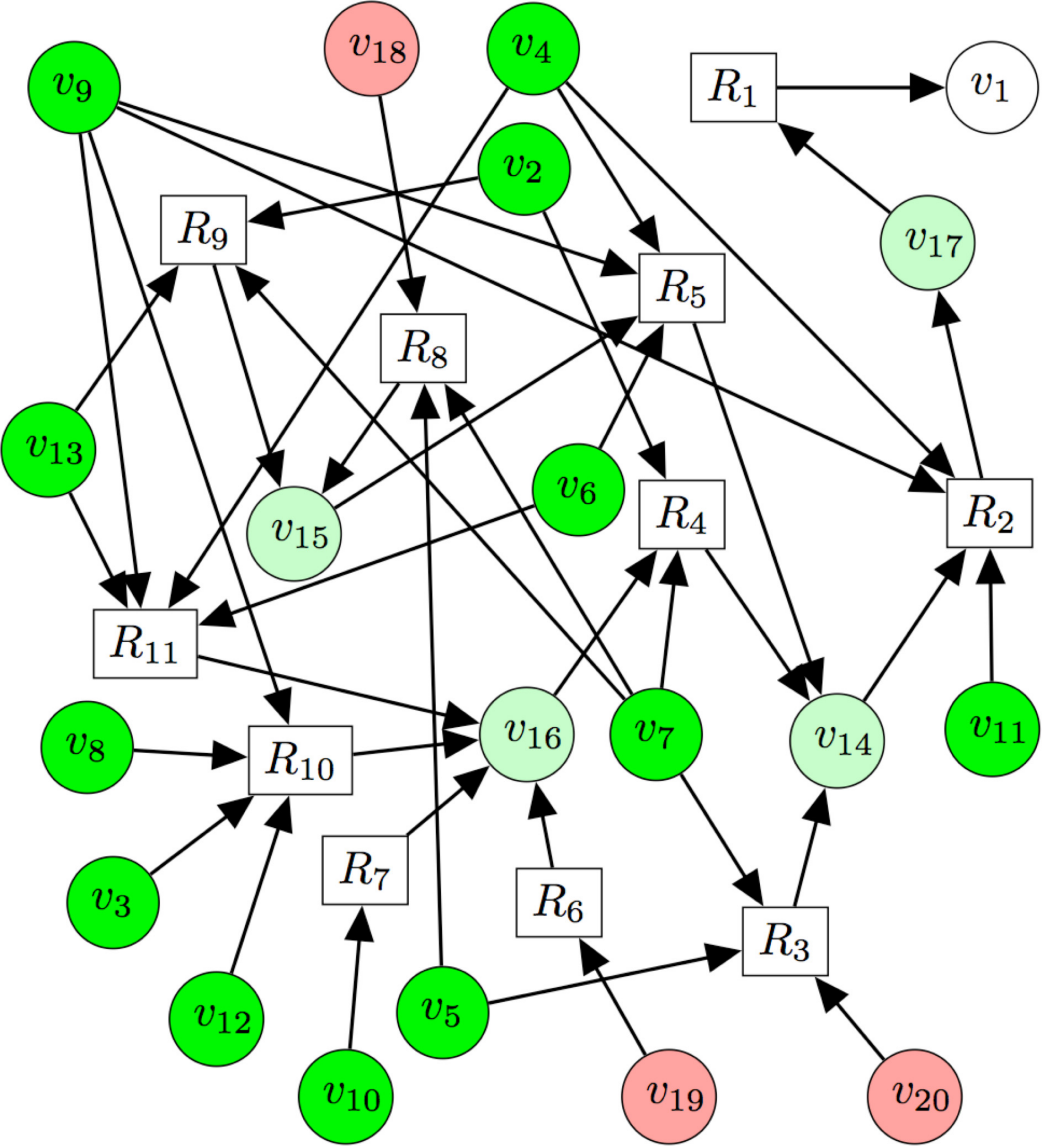
**Known heterologous reactions leading to the production of liquiritigenin (white circular node)**. Squares represent reactions and circular nodes represent molecules. Dark green nodes are present in the host organism, *E*. coli K-12, light green can be produced via enzyme catalyzed reactions and red ones cannot. Liquiritigenin, a highly selective estrogen receptor *β *agonist, is represented by the white node *v*_1_. Side products not consumed in this pathway are not represented for simplicity. Four pathways lead from the host compounds to the production of the target molecule, one for instance is the pathway involving reactions *R*_1_, *R*_2_, *R*_4_, *R*_7_. Legend: *v*_1_: liquiritigenin, *v*_2_: ATP, *v*_3_: NADH, *v*_4_: NADPH, *v*_5_: NADP+, *v*_6_: oxygen, *v*_7_: CoA, *v*_8_: acetate, *v*_9_: H+, *v*_10_: L-tyrosine, *v*_11_: malonyl-CoA, *v*_12_: 4-hydroxybenzoate, *v*_13_: trans-cinnamate, *v*_14_: p-coumaroyl-CoA, *v*_15_: cinnamoyl-CoA, *v*_16_: 4-coumarate, *v*_17 _isoliquiritigenin, *v*_18_: cinnamaldehyde, *v*_19_: 3-(4-hydroxyphenyl)lactate, *v*_20_: 4-hydroxycinnamyl aldehyde.

The aim of the work presented here is to find all the viable sets of heterologous enzymes, which can produce a predefined target compound when added to the pool of endogenous enzymes of a given organism. Our method enables a metabolic engineer to find all heterologous metabolic pathways producing a target compound, for instance liquiritigenin (a plant secondary metabolite with therapeutic applications), from the endogenous metabolites of *E*. coli K-12, as shown in Figure 1.

As depicted in Figure 1, our problem can be formulated as searching for all possible heterologous pathways linking a target compound to the endogenous metabolites of an organism. To this purpose we provide software tools that enable the discovery of potential pathways producing a target chosen by the user [1]. More precisely the user enters a target compound, a chassis organism, and our software tools return a ranked list of pathways (each list being composed of enzymes) to be engineered into the chassis organism. To achieve this task we have developed an approach composed of three steps. In the first step, using a retrosynthesis software, reactions producing a target compound are iteratively searched backwards until the set of needed precursors only contains source metabolites. This first step returns a retrosynthetic network connecting a target compound to the endogenous metabolites of an organism. There may be several pathways in the retrosynthetic network linking the source metabolites to the target compounds and there is thus a need to enumerate all the possibilities. Pathway enumeration is performed by in the second step. Once the pathways have been enumerated, we evaluate in the third step the possibility to insert each pathway and its associated heterologous enzymes in the host organism. This step consist of determining the catalytic efficacy of the enzymes, the toxicity of the products and the coproducts [2], and the easiness of inserting the enzymes into the host. The efficiency of the pathways can then be further estimated by flux models for the cell metabolism such as flux balance analysis [3].

We have already discuss elsewhere the first and third step [1,2], i.e. methods to generate retrosynthetic networks and methods to rank pathway efficiency. To apply these methods in the context of heterologous target production, we need a computationally fast method to enumerate all possible pathways. We address the enumeration problem in the current paper.

Different mathematical models that describe metabolism have been proposed (cf. [4] for a review of the different models). We distinguish two main families of approaches: the ones computing steady states of the fluxes of reactions (one well-known application being the flux balance analysis) and the ones based only the topology of the network. Typically, steady states are studied and simulated by generating the flux space. Of particular interest are the extreme pathways and the elementary modes, they both represent the smallest (minimal) generating set of the flux space and they both are composed of independent non-decomposable pathways in the network [4]. The differences between extreme pathways and elementary modes have already been discussed in details [5] and these differences arise when dealing with reversible reactions. In the present paper we consider all reaction irreversible, and networks comprising reversible reactions are modeled by doubling each reversible reaction into a forward reaction and a reverse reaction. Algorithms have been developed to enumerate both extreme pathways [6] and elementary modes [7] and these algorithm are all variants of the double description method [8], which enumerates all extreme rays of a polyhedral cone. The algorithms use as input a stoichiometric matrix (**S**) representing the network (cf. [3] for definition of stoichiometric matrix) and output sets of fluxes (**v**) satisfying **Sv **= 0. One notices that extreme pathways and elementary modes while representing pathways (to each flux verifying **Sv **= 0 correspond a stoichiometrically balanced pathway) do not directly enumerate all pathways linking a source set to a target set of compounds. However as shown in the subsection "Enumerating pathways using the steady state approach" one can construct stoichiometric matrices where input fluxes are added to the set of source compounds and outgoing fluxes are associated to the target and heterologous coproducts such that the extreme pathways and elementary modes enumerated from these matrices do correspond to all pathways linking the source set to the target.

While as mentioned above, the problem of systematically enumerating pathways for heterologous production in chassis organisms has not yet been addressed, there are methods based on the steady state approach to search for heterologous pathways optimizing target productions [9], and methods to search for shortest pathways between source and target sets of compounds [10] and [11]. All these methods are based on optimization and make use of integer linear programming. Precisely, the method of Pharkya *et al*. [9], is aimed at redesigning microbial chassis organisms through heterologous reaction addition and native reaction deletion for the overproduction of a target compound. The addition and deletion are parameterized using binary variables attached to each reaction. A mixed integer linear program (MILP) is then set up to maximize the target yield while minimizing the number of added reactions. The methods of de Figueiredo *et al*. [10], and Pey *et al*. [11] are both aimed at searching for the *k *shortest pathways. In de Figueiredo *et al*. [10] the *k *first shortest pathways are searched in entire metabolic networks, while in Pey *et al*. [11] the pathways are searched between a source metabolite and a target metabolite. Both methods solve the problem at steady state and search for fluxes, *v*, verifying **Sv **= 0, while minimizing the number of reactions turned on (using a binary variable). Aside from the fact that integer linear programs suffer from computational complexity (MILP is an NP-hard problem) all the above methods search for at most *k *optimized (shortest) pathways and do not guarantee a full enumeration of the possibilities. In our methods the optimal pathways are computed in a post process by ranking the pathways that have been enumerated. Our approach allows one to decouple enumeration from optimization, and thus to plug any optimization criteria, including nonlinear functions and not only target yield or pathway length (cf. page 3 and Carbonell *et al*. [1] for a list of criteria entering our metabolic engineering optimization problem).

Aside from using extreme pathways and elementary modes, we also present in this paper a topological model which directly enumerates all the possible heterologous pathways linking target compounds to a source set of compounds. The main advantage of the topological approach compared to the stationary state approach is computational speed. Speed is in fact an important aspect when searching for the best pathways to engineer, as there are generally a combinatorial number of pathways between given source sets and target sets. As an illustration of this combinatorial complexity, the work of Hatzimanikatis et *al*. [12], which provides a list of 75,000 novel biochemical routes from chorismate to phenylalanine, and the work of Cho et *al*. [13], which enumerates 107,272 reaction routes to produce isobutanol.

There exist standard graph-based methods to search and eventually enumerate pathways in metabolic networks, but these methods including PathFinding [14-16] and Pathway Hunter Tool [17] are computing pathways and shortest pathways in graphs instead of hypergraphs. The particularity of these techniques is that only main substrates and main products are taken into account when constructing pathways, and consequently these main compounds must be differentiated from the cofactors (i.e. co-substrates and co-products). In the work of Croes *et al*. [14,15] cofactors are filtered out based on their connectivity in the network. Indeed, compounds highly connected such as ATP, NADP, or H_2_O are cofactors of most reactions as they do not share carbon atoms with the products of the reactions. In a more recent work [16], the main compounds in the pathways linking source metabolites to target metabolites are detected using the Kegg RPAIR annotation [18,19], which enables one to follow the fate of atoms when going from a set of substrates to a set of products. Another approach to search for main substrates and main product is the one developed with the Pathway Hunter Tool, which consists of mapping substrates to products using cheminformatics fingerprints. While all the above techniques are computationally efficient, their main shortcoming is that they are not able to encompass reactions when a main product is formed from two main substrates. There are plenty of such reactions in metabolic networks, consider for instance the formation of guanidinoacetate from arginine and glycine through a glycine amidinotransferase (EC 2.1.4.1), or the formation of glutathione from *γ*-L-glutamyl-L-cysteine and glycine catalyzed by a glutathione synthase (EC 6.3.2.3). Recently, some of the above topological methods have been benchmarked against the integer linear programming technique mentioned above [11] to search for the shortest pathways linking various compounds, the recovery ratio for a set of 40 predefined reference pathways could not reach 100% with the graph based approach, exemplifying the shortcoming of that approach.

As reviewed above, while there are methods and theoretical results to enumerate elementary modes or extreme pathways and graph based techniques to search for pathways in a given metabolic network, to the best of our knowledge there is no known methods to directly enumerate pathways in the context of metabolic engineering, that is, to enumerate all the pathways encompassing all substrates and products necessary and sufficient to produce a given set of target compounds from a given set of source compounds. In the present paper we address that specific problem and present two methods one based on elementary modes (steady state approach) and one based on a direct enumeration algorithm (topological approach). In order to address this problem we need, in addition, to consider the problem of determining supplement molecules, i.e. metabolites that the organism cannot synthesize, but which can be added to the growth media in order to increase the number of viable pathways; and bootstrap molecules, i.e. metabolites which are required fist in order to be produced [20]. While in the general context of metabolic network analysis, finding elementary modes does not require to first search for bootstrap molecules, in the context of metabolic engineering however any heterologous pathway solution that comprises a compound that is first consumed before being produced is valid only when the compound is added to the growth medium. Therefore, in our study in the context of metabolic engineering, there is a need to first compute the bootstrap compounds prior to elementary modes.

The paper is divided as follows. In the Methods section we first provide some definitions, then outline our algorithms to solve the pathway enumeration problem with both the steady state approach and the topological approach. The problem of finding and enumerating all the pathways going from a large source (as for instance al the metabolites of an organism) to a target chosen by the user is considered. All the algorithms presented for the topological approach (with the exception of the algorithm for enumeration) have polynomial worst-case running time, the algorithm for enumeration is a polynomial time per output algorithm on some classes of hypergraphs. We also provide algorithms to determine supplements, which are metabolites that the organism cannot synthesize, but which can be added to the growth media in order to increase the number of viable pathways. Furthermore, an analysis of pathways containing supplements allows finding out pathways that contain bootstrap molecules. In the Results and Discussion section we illustrate our algorithms with the enumeration of the possible pathways to synthesize more than 5000 compounds in *E. coli*. We "experimentally" probe the computational complexity of the steady state and topological approaches for a series of networks of growing sizes and discuss the theoretical complexity results of the topological approach, which are provided in Appendices A and B.

While we illustrate our two methods for the production of heterologous compounds using as a source set all the endogenous metabolites of *E*. coli, our methods can be applied to any chassis organism and more generally to any source set (for instance a set of nutrients or a set of abundant currency metabolites).

## Methods

In the context of metabolic engineering, metabolic networks have been represented as directed graphs (cf. for instance Cho *et al *[13]). In such graphs, edges are directed and correspond to reactions connecting two compounds if one is the product of the other. Directed graphs can represent monomolecular reactions (one substrate gives one product), but they are not well suited to capture more complex reactions. As already discussed in the background section, when representing bimolecular reactions, one has to choose which molecules are connected by the edges of the graph and which ones have to be excluded from the graph because they are considered co-substrates or co-products. Additionally, one of the limitations of a model based on a graph representation is that depending on the criteria used to identify the co-substrates and co-products in the reactions, the networks obtained are different.

In the present paper to palliate the limitations of the directed graph model, we represent networks as directed hypergraphs. The first examples of modeling through hypergraphs can be found in [20]. In a hypergraph, each hyperarc connects a set of vertices, corresponding to reactants, to a disjoint set of vertices, representing the products. In our model each hyperarc corresponds to a reaction that can be catalyzed by an enzyme. It is worth noticing that hypergraph models have already been used to find minimal sets of metabolites sufficient to produce a set of target metabolites [21]. Unfortunately, the algorithms proposed in [21], do not enumerate pathways and are therefore not directly applicable to our metabolic engineering problem.

## Definitions

**Definition 1 **(Hypergraphs and hyperarcs).

*A directed hypergraph is a pair H=(V,E) where V *= {*v*_1_, *v*_2 _..., *v_n_*} *is the set of vertices and E *= {*e*_1_, *e*_2_,..., *e_m_*} *is the set of hyperarcs. A hyperarc e_i _is an ordered pair e_i _*= (*X_i_*, *Y_i_*) *of disjoint subsets of V*.

The set *X_i _*is also called the tail of *e_i _*and the set *Y_i _*is called the head, with reference to the graphical representation of arcs (directed edges) and hyperarcs as arrows.

We denote by X:E→P(V) the application that given an hyperarc *e_i _*returns its tail *X*(*e_i_*) ⊂ *V*. Analogously we use Y:E→P(V) for the application that given a hyperarc returns its head.

**Definition 2 **(Reactions and networks).

*In a metabolic network each vertex corresponds to a metabolite and each hyperarc corresponds to a reaction. A metabolic network of m metabolites and n reactions can be represented with a m *× *n stoichiometric matrix ***S***, where the rows correspond to the m metabolites and the n columns to the reactions. A reaction j is represented by the column vector S_j _*= (*s*_1*j*_,..., *s_mj_*)*^T ^where s_ij _is the stoichiometric coefficient of metabolite i in reaction j. Reactants have negative coefficients and products have positive coefficients*.

**Figure 2:**
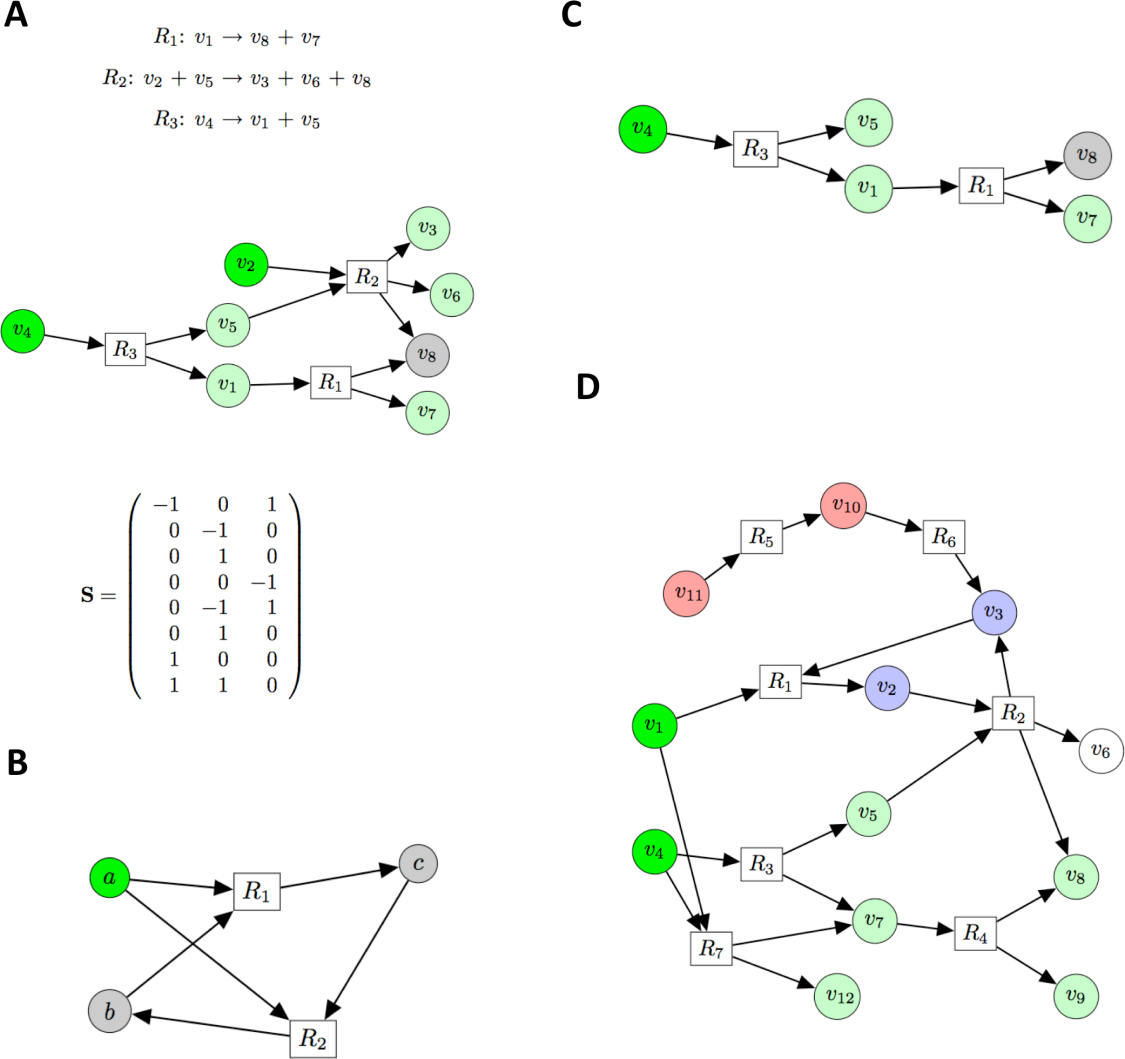
**Reactions, hyperpath, and corresponding stoichiometric matrix**. A) A set of reactions (top), the corresponding hypergraph, and the corresponding stoichiometric matrix (bottom). The hypergraph here represented is a hyperpath from *v*_2 _and *v*_4 _(source nodes) to the target vertex *v*_8_. The reactions can be ordered *R*_3_, *R*_2_, *R*_1 _so that the conditions required by the hyperpath definition (3) are satisfied; B. A hypergraph that is not a hyperpath: The hypergraph {*R*_1_, *R*_2_} is not a hyperpath with source *a*; C) A minimal hyperpath: This hyperpath is a subset of the hyperpath in Figure 2A and is minimal (both *R*_1 _and *R*_3 _are necessary to link *v*_8 _to the source); D) A hypergraph representing a toy metabolic network: Given *v*_1 _and *v*_4 _as sources of the hypergraph above the reachable vertices are *v*_5_, *v*_7_, *v*_8_, *v*_9_, *v*_12 _in light green. *v*_2 _and *v*_3 _are bootstrap compounds: the presence of one of them permits its own production. In red the compounds *v*_10 _and *v*_11 _are supplements for the production of *v*_2_, *v*_3_, *v*_6 _and *v*_8_.

Examples of hypergraph, network, and stochiometric matrix are given in Figure 2A. We notice that the stoichiometric coefficients of the reactions are not taken into account in the hypergraph representation. We also notice that the pair (*X*, *Y*) is ordered so to make the distinction between reactants and products. In this representation reactions are irreversible. Many biochemical reactions can be considered as irreversible, since in organisms the homeostatic equilibrium is often strongly polarized. Nonetheless, metabolic network may comprise reversible reactions, and we model these reactions by introducing both hyperarcs: (*X*, *Y*), and (*Y*, *X*).

Hyperpaths, a generalization of simple paths in graphs where cycle free paths going from one vertex to another, are used to represent pathways. A hyperpath connects a source set of vertices to a target set of nodes. Two examples of hyperpaths are given in Figures 2A and 2C. We remark that in a natural way a set *E *of hyperarcs defines a hypergraph *ε *= (∪_*e*∈*E*_*X*(*e*) ∪ ∪_*e*∈*E*_*Y *(*e*), *E*). By abuse of the terminology we denote by *E *the hypergraph corresponding to the set *E *of hyperarcs and all the heads and tails of the hyperarcs in *E*. The following definition for hyperpaths is borrowed from Nielsen et *al*. [22].

**Definition 3 **(Hyperpaths).

*A hyperpath P going from a source subset $S_{H}$ of V to a target subset T_P _of P in a hypergraph H=(V,E) is a hypergraph $H_{P}=\left(V_{P},E_{P}\right)$ with VP *⊆ *V, EP *⊆ *E, such that there is an ordering **F **of the hyperarcs EP with the following properties*.

• $\forall k\in \left\{0,\ldots ,|F|\right\},X\left(F_{k}\right)\subseteq S_{H}\cup \left(\cup_{j<k}Y\left(F_{j}\right)\right)$

• $T_{P}\subseteq S_{H}\cup \left(\cup_{e\in E_{P}}Y\left(e\right)\right)$

From the point of view of metabolism, the first condition corresponds to the requirement that reactants of reactions participating in the hyperpath can be produced without the presence of the reaction itself. Hyperpaths defined in this manner represent a metabolic route from the source to the target. According to definition (3) the hypergraph of Figure 2B with source *a *is not a hyperpath because neither reaction *R*_1 _nor *R*_2 _can happen until the other does not start. The definition (3), though complex, is computationally tractable, meaning that the time required to determine if a hypergraph is a hyperpath is proportional to the number of reactions. A polynomial time algorithm to determine if a hypergraph is a hyperpath is given in [23], the algorithm *FindAll *presented below can also be used for that purpose. In fact, as discussed below, if the set of reactions returned by *FindAll *$\left(H_{P},S_{H}\right)$ contains all the reactions in $H_{P}$, then $H_{P}$ is a hyperpath.

The metabolic network described by a hypergraph has to be as comprehensive as possible, containing every known enzyme-catalyzed reaction occurring in organisms. We say that a hyperpath produces a set of target metabolites if it contains all those target elements. A set of target compounds is said to be reachable from a given source, or linked to the source, if there is at least one hyperpath producing the targets.

We are interested in the enumeration of pathways leading to the production of a desired compound. Hyperpaths do not generally give the best representation of pathways because hyperpaths can contain reactions not necessarily linking the target to the source. Minimal hyperpaths, cf. definition (4), are an appropriate representation of pathways since they contain only the essential reactions linking the source to the target.

In the definition given below, we say that a hyperpath P(V,E) is a subset of another hyperpath $P^{\prime }\left(V^{\prime },E^{\prime }\right)$ if *V *⊆ *V' *and *E *⊆ *E'*. For instance the hyperpath of Figure 2C is a subset of the one of Figure 2A.

**Definition 4 **(Minimal Hyperpaths).

*A hyperpath *(*V_P_*, *E_P_*) *with target TP is said to be minimal if it has no proper subsets with the same target*.

The target is disconnected from the source if a reaction is removed from a minimal hyperpath. In this sense minimal hyperpaths cannot be reduced. From a metabolic engineering perspective the concept of minimal hyperpath is useful as it defines the minimum set of reactions necessary to produce a target heterologous compounds, and consequently the minimum set of enzymes needed to be inserted into the chassis organism where the compound is going to be produced.

In the following we define $B\left(H,S_{H}\right)$ to be the set of all molecules linked to the source for a given hypergraph H and source set $S_{H}$. The characterization of $B\left(H,S_{H}\right)$ is the first task to be solved before the enumeration. Once this set is known all the minimal hyperpaths can be enumerated for all the molecules associated to the vertices in $B\left(H,S_{H}\right)$.

### Supplements

Supplements for a target are molecules whose presence in the source set increases the number of pathways for target production. Finding supplements is an important improvement when exploring ways to produce the target, since they make possible new pathways.

For each target of interest one can look for vertices that once inserted in $S_{H}$ give place to pathways otherwise impassable. In terms of metabolism we are looking for the "supplement" molecules, i.e., molecules that once introduced in the source set permit to find more pathways than those otherwise available. We introduce below *FindSupp*, an algorithm that returns the supplements.

An analysis of pathways containing supplements allows to find out pathways containing bootstrap molecules, i.e. metabolites that are needed in reactions producing compounds afterwards used for the production of the bootstrap molecules. As a matter of fact, many pathways can be made viable once bootstrap molecules become available in the metabolic network (a concept introduced in [20]). Loosely speaking bootstrap molecules are molecules that cannot be produced by the reactions belonging to a hyperpath unless they are already present in the source. Cottret *et al *[21] stated that given a source set the existence of a pathway making use of bootstrap molecules can be tested in polynomial time. We provide later in this section an algorithm returning the bootstrap compounds, such algorithm can be used to determine if a target molecule is connected to the source through a pathway making use of bootstraps.

### Enumerating pathways using the steady state approach

In steady state, all possible pathways in a metabolic network are by definition stoichiometrically balanced, i.e. all metabolites produced from the source set must be consumed except for those that are target products. Extreme pathways and elementary modes are two methods that compute the set of independent non-decomposable pathways in the network that generate all feasible steady state solutions in the flux space. They do not directly enumerate all pathways linking a source set to a target set of compounds. However, one can construct stoichiometric matrices where input fluxes are added to the set of source compounds and outgoing fluxes are associated to the target and heterologous co-products such that the extreme pathways and elementary modes enumerated from these matrices can be used to generate all pathways linking the source set to the target.

Given a hyperpath $H_{P}=\left(V,E\right)$ of a hypergraph H=(V,E), we can define a set of flux vectors **v***_P _*for the hyperpath where components *v_Pj _*corresponding to those reactions in the pathway $e_{j}\in H_{P}$ are activated:

(1) $$v_{Pj}=\;\left\{\begin{array}{cc} >0 & e_{j}\in H_{P}\\ 0 & e_{j}\in H\backslash H_{P} \end{array}\right)$$

A hyperpath $H_{P}=\left(V,E\right)$ of a hypergraph H=(V,E) with input source subset $S_{H}$ and the target subset *T_P _*is defined as stoichiometrically balanced if the rows corresponding to each metabolite *v_i _*∈ *V *that are obtained from the product of the stoichiometric matrix **S **and the associated flux vector **v***_p _*verify:

(2) $$Sv_{P}=\left\{\begin{array}{cc} \leq 0 & v_{i}\in S_{H}\\ \geq 0 & v_{i}\in T_{P}\\ 0 & v_{i}\in V\backslash \left\{S_{H},\;T_{P}\right\} \end{array}\right)$$

A way to introduce the constraint on input and output metabolites in the previous equation is by adding to the stoichiometric matrix **S **additional columns corresponding to input reactions (reactions with no substrate that produce the source set $S_{H}$), and output reactions (reactions with no product that consume the product metabolites in the hypergraph *T_P_*). These auxiliary reactions, even if non-properly balanced in terms of the law of conservation of mass, are useful in order to define completely the problem in a compact manner:

(3) $$\begin{array}{cc} Sv=0 & \\ v\geq 0 & v\in R \end{array}$$

Both extreme pathways and elementary modes make use of this formulation in order to compute the set of feasible solutions **v**. Since in our hypergraph definition all reactions are irreversible, the set of pathways solving Equation 3 computed by both extreme pathways and elementary modes are identical (cf. [5]). Furthermore, solutions in **v **must contain only positive or null fluxes.

In order to determine all stoichiometrically balanced heterologous pathways $H_{P}$ that can be inserted into the chassis organism to produce a target set *T_P_*, we need to constrain the computation of elementary modes only to those that have non-zero fluxes for heterologous reactions. Efficient solutions to this problem have been considered in the divide-and-conquer approach [24,25] by rearranging the constraints in an echelon form so that the constraints containing only the desired reactions appear at the bottom. To define the constraints in our case, we consider first the hypergraph $R_{T}$ that is formed only by heterologous reactions. This hypergraph $R_{T}$ is the subset of the hypergraph R(V,E) formed by those hyperedges where at least one vertex *V *does not belong to the source set $S_{R}$, i.e. those metabolites endogenous to the chassis organisms. By considering $R_{T}$ instead of the full hypergraph R, we are looking only for biosynthetic pathways involving heterologous reactions and therefore avoiding cycles internal to the chassis organism. Therefore, to compute all feasible steady state heterologous pathways, we reformulate Equation 2 so that the stoichiometric matrix **S **is defined by reactions in $R_{T}$; the input is given by all substrates in the source set $S_{R}\cap X\left(E_{R}\right)$; and the output by all products of the reactions in the hypergraph $Y\left(E_{R}\right)$.

Finally, from the computed set of solutions **v **for Equation 3, we are interested in enumerating all minimal hyperpaths from $S_{R}$ to the target set *T *on the hypergraph given by $R_{T}$. According to Definition 4, minimal hyperpaths for some target *T *are given by those cycle-free solutions in **v **containing only reactions linking the source to the target. Since any feasible flux pattern **v **is a superposition of elementary modes with non-negative coefficients [26], the set of minimal hyperpaths for a given target *T *is a subset of the elementary modes producing *T *that are solution of Equation 3. Namely, any feasible solution generated from the elementary modes will contain at least as many reactions as the ones that are in those elementary modes that form its basis. Therefore no additional minimal hyperpaths can be generated in this case by superposition of elementary modes.

### Enumerating pathways using the topological approach

The algorithm *FindAll *that allows to find $B\left(H,S_{H}\right)$, the set of metabolites that can be linked to the source $S_{H}$ by a hyperpath. *FindAll*, by explicitly constructing the ordered set ***F ***in definition (3), provides a proof of the tractability of the problem of checking if a hypergraph is a hyperpath. Moreover *FindAllF *permits to prune the original hypergraph enabling a faster enumeration algorithm.

As presented below the algorithm *Minimize*, when called on the output of *FindAll*, returns, if exists, a minimal hyperpath linking a given target to the source. These algorithms are the main components of the algorithm enumerating the pathways *FindPath *described next. Then we present *FindSupp *an algorithm to enumerate supplements.

#### Finding one minimal hyperpath

Let H=(V,E) be the hypergraph representing the set of metabolic reactions, *n *= |*V|*, *m *= |*E| *and let $S_{H}$ be the set of source vertices representing the source metabolites.

The algorithm *FindAll *returns all the reactions that can contribute to the production of any element in $B\left(H,S_{H}\right)$, i.e., the set of all compounds that can be connected to the source. *FindAll *is a linear algorithm in the number of vertices, hyperarcs and in the total coordination; the complexity is *O*(*n *+ *m *+Σ_*v*∈*V *_|*X *^-1^(*v*) |+ |*Y*^*-*1^(*v*)|) that is bounded by *O*(*n *+ *m *+ *n · m*). Therefore, such algorithm can be applied to the hypergraph H of all reactions in order to obtain a pruned sub-hypergraph $H^{\prime }=\left(V^{\prime },E^{\prime }\right)$ where the set of vertices $V^{\prime }:=S_{H}\cup B$, and the set of edges *E' *is the set of reactions returned by *FindAll*. In the context of metabolic engineering *FindAll *returns all the compounds that can be produced from a given set of source compounds and reactions. For instance, using *FindAll *with all know metabolic reactions one can determine all the compounds that can be produced from the metabolites of *E*. coli.

**Algorithm FindAll **(Given a hypergraph H and a source $S_{H}$, returns all the hyperarcs that are part of at least one hyperpath.)

input:

$H,S_{H}$

1. **for all ***r ***in H**

2.    *x*(*r*) ← *X*(*r*)

3. **end for**

4. $V\leftarrow S_{H}$

5. $D\leftarrow S_{H}$

6. ***F ***← {∅}

7. **while ***V *≠ {∅}

8.    let *i *be an element of *V*

9.    *V *← *V \ i*

10.    *D *← *D *∪ *i*

11.    **for all ***r *∈ *H *such that *i *∈ *x*(*r*):

12.       *x*(*r*) ← *x*(*r*) *\ i*

13.       **if ***x*(*r*) = {∅}

14.          ***F ***← {***F***, *r*}

15.          **for all ***j ***in ***Y *(*r*) **and not in ***D*:

16.             *V *← *V *∪ *j*

17.          **end for**

18.       **end if**

19.    **end for**

20. **end while**

output:

   ***F***

Let *D *be the union of the source set and of the heads of all the reactions output in ***F ***by *FindAll*. The correctness of the algorithm above is given by the following claims: every element in *D *is the target of some hyperpath or is part of the source, and every vertex in H that can be reached from the source is in *D*. For the first claim we can give a constructive proof by using the output vector ***F***, the second claim is proved by contradiction.

• The proof of the fact that every element in *D *is reachable from the source is given constructively by the ordered set ***F ***returned by the algorithm. In fact at each step ***F ***is a hyperpath. This claim can be proved by induction on the steps of the algorithm, each time a hyperarc *r *is appended to ***F ***(line 14) the tail *X*(*r*) is contained in *D *(hyperpath by inductive hypothesis) and if a vertex *j *is added to *D *(line 10) it means that it was previously added to *V *(line 16) and thus it was in the head *Y *(*E*) of some hyperarc already in the hyperpath.

• The second claim can be proved by contradiction: if an element of $B\left(H,S_{H}\right)$ were not in *D *there would be a hyperpath linking it to the source. In such hyperpath let consider the first (according to the order given by the definition) reaction *r *whose *X*(*r*) belongs to *D *and such that one of the elements of *Y *(*r*) does not. Consider among *x*(*r*) the last one that has been inserted into the set *V *; after its removal from *X*(*r*) this set becomes empty and the elements of *Y *(*r*) are inserted into *V *(line 16) and then in *D *(line 10), which is a contradiction.

From the above statements follows that each vertex appearing in a hyperpath having as source $S_{H}$ is an element of *D *and every hyperarc is an element of ***F***. Thus the algorithm *FindAll *provides an effective pruning of the original hypergraph: in H there is no minimal hypergraph with source $S_{H}$ containing hyperarcs not in ∪*_k_F_k _*or vertices not in $B\left(H,S_{H}\right)$. The output hyperarcs are the only ones that can belong to a minimal hyperpath, and $H^{\prime }=\left(S_{H}\cup \left(\cup_{k}Y\left(F_{k}\right)\right),\cup_{k}F_{k}\right)$ is the pruned hypergraph only containing reachable vertices and hyperarcs.

Notice that *FindAll *algorithm as presented above returns in polynomial time a hyperpath valid for each target vertex in $B\left(H,S_{H}\right)$. Even though there are more efficient algorithms for finding a hyperpath for one single target, for the sake of simplicity we avoid to introduce here an additional algorithm and just remark that since *FindAll *is polynomial, the use of it does not affect the complexity analysis of the algorithms making use of its output.

Remark that a minimal hyperpath going to a specific target can be easily extracted from the hyperpath output of *FindAll*. Namely, given a hyperpath P connecting *S *to *T*, it is always possible to find a minimal hyperpath $P^{\prime }$ subset of P. Moreover it can be done in polynomial time, for instance by using *Minimize *(P,{∅},T,S), the algorithm introduced below.

*Minimize*$\left(P,R_{f},T,S\right)$ is an algorithm that takes as input a hypergraph P, a hyperpath *R_f _*subset of P, a target set of vertices *T *and a source *S*. If P does not link *T *to *S *the empty set is returned, otherwise a hyperpath contained in P, containing *R_f _*and linking *T *to *S *is returned. In particular, if *R_f _*is empty, the output of *Minimize *is the minimal hyperpath going from *S *to *T*, provided it exists. *Minimize *returns a hyperpath obtained by removing all inessential hyperarcs except for the ones in *R_f_*. In the context of metabolic engineering, pathways containing a small number of heterologous reactions are generally preferred, since they are easier to engineer in the host organism. Therefore, given two pathways that produce the same target, where one is subset of the other, the one requiring the smaller number of heterologous reactions has to be selected. This is the reason that makes relevant to obtain minimal hyperpaths from generic hyperpaths.

**Algorithm Minimize **(Given a hypergraph P containing *R_f_*, returns either a hyperpath from *S *to *T *containing *R_f _*or an empty set if *T *is not linked to *S *by P.)

input:

$P,R_{f},T,S$

1. ***F ***← FindAll(P, *S*)

2. *P' *← *P*

3. **if ***T *⊄ *∪_k _Y *(*F_k_*)

4. $P^{\prime }\leftarrow \left\{\emptyset \right\}$

5. **else**

6.    **for all ***r ***in P**

7.       **if ***r ***not in ***R_f_*

8.          ***F ***← FindAll($P^{\prime }$*\r*, *S*)

9.          **if ***T *⊂ ∪*_k _Y *(*F_k_*)

10.             $P^{\prime }\leftarrow P^{\prime }\backslash r$

11.          **end if**

12.       **end if**

13.    **end for**

14. **end if**

output:

   $P^{\prime }$

The proof of correctness of this algorithm is simple and is based on the fact that $P^{\prime }\subseteq P$ implies $FindAll\left(P^{\prime },S\right)\subseteq FindAll\left(P,S\right)$. If a reaction in P has not been removed from $P^{\prime }$, then any subset of *P^0 ^*not containing *r *does not produce the target. The worst-case time for this algorithm is $O\left(m\cdot \left(n+m+\sum_{r\in P}|X\left(r\right)|+|Y\left(r\right)|\right)\right)$. Since X(r) and Y(r) have bounded values, the algorithm has a quadratic complexity. Even though faster algorithms can be designed, here we presented this one because of its conceptual simplicity. Remark that, since *Minimize*(P, {∅} *T*, *S*) returns a minimal hyperpath $P^{\prime }$ subset of P if it exists, then the minimality of a hyperpath P can be tested by checking whether $P^{\prime }=P$ or not.

A related problem to *Minimize *is the minimal constrained hyperpath problem: the problem of finding if a minimal hyperpath from a given source to a given target, containing the hyperarcs in *R_f _*exists. Notice that *Minimize*, although linked to this problem does not solve it. In fact, if the output of *Minimize *is an empty set then there are no minimal hyperpaths satisfying the constraints; however if the output is a minimal hyperpath then obviously a minimal constrained hyperpath exists; and finally, if the output is a non-minimal hyperpath then we do not know if a minimal hyperpath satisfying the constraints exists or not.

Below we will discuss why we are interested in algorithms for the minimal constrained hyperpath problem, while in Appendix A.2 we show that in general the problem is NP-complete (reduction to 3-SAT).

#### Pathways Enumeration

The basic idea behind the enumeration algorithm presented below is to introduce an iterative refinement of partitions of the space of feasible solutions i.e. of the space of hyperpaths and in each part to look for a solution. In our implementation, a part is defined by two sets of reactions (*R_f _*and *R_n_*) of the original hypergraph. These sets are used during the enumeration process, *R_f _*is a set of hyperarcs that must be present in the enumerated hyperpath and *R_n _*is the set of hyperarcs that must not be part of the enumerated hyperpath. The problem of finding a solution in one of the parts is addressed at each iteration and if a solution is found the part is divided in finer parts. This process is repeated until all the minimal hyperpaths have been found.

#### Enumeration by means of the minimal constrained hyperpath problem

**Figure 3:**
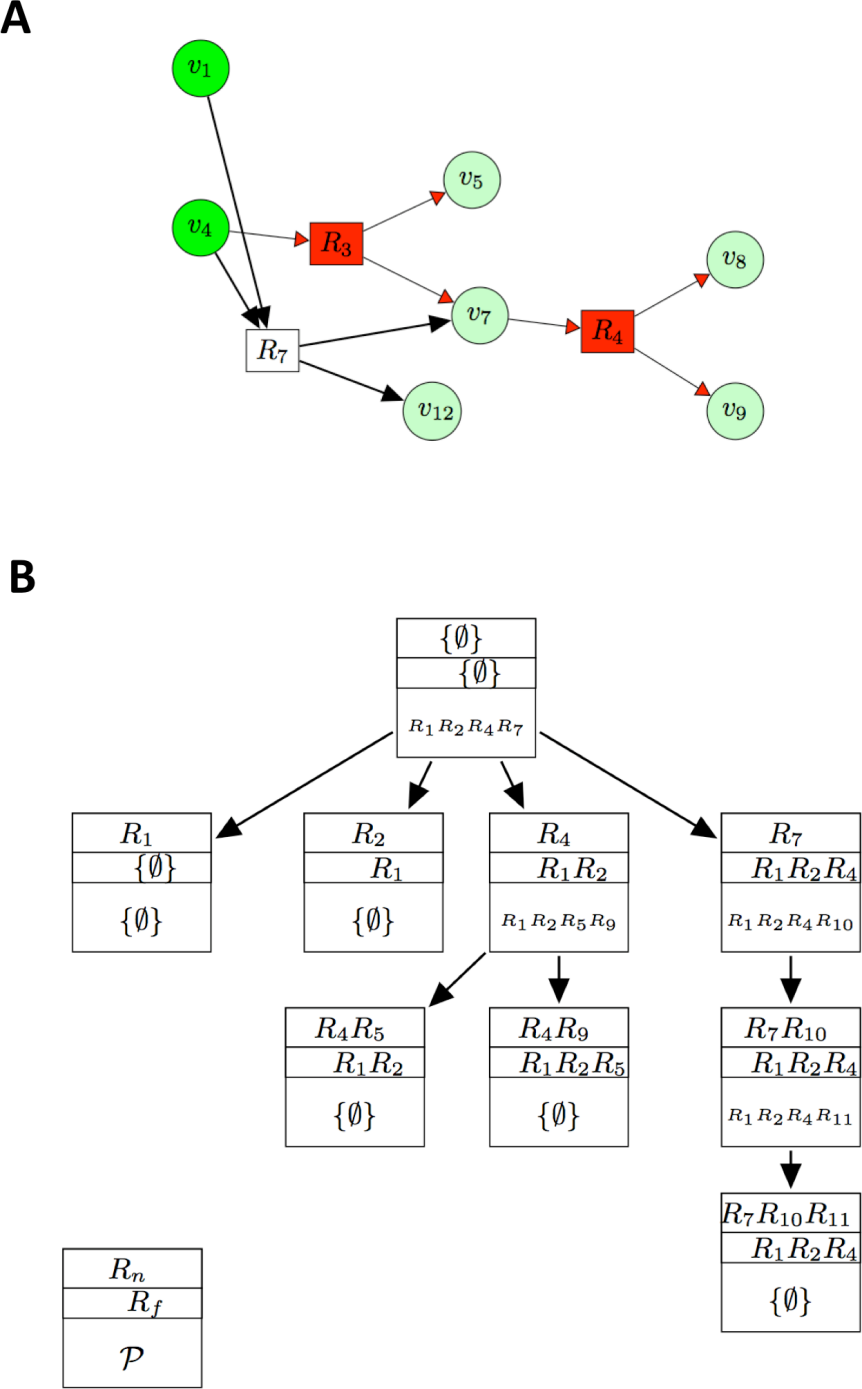
**The resolution of the enumeration problem**. A) The process of enumeration: The hyperpath given by running *FindAll *on the hypergraph of Figure 2D. This hyperpath is not minimal as can be verified by running *Minimize *without constraints (i.e. *R_f _*= {∅}). Two minimal hypergraphs connect *v*_8 _to the source nodes *v*_1_, *v*_4_: the one containing hyperarcs *R*_4_, *R*_7 _and the one highlighted in the figure containing only the hyperarcs *R*_3_, *R*_4_; B) Scheme of resolution of the enumeration problem for biosynthesis of liquiritigenin whose hypergraph is represented in Figure 1. Each node in the scheme corresponds to a call to *FindPath *and contains the constraint sets *R_f _*and *R_n _*(see left bottom box), and the minimal hyperpath P corresponding to the given constraints. *FindPath *iteratively calls itself, the structure obtained is a rooted tree where each node represents a call to *FindPath*, and each call is characterized by the sets *R_f _*and *R_n_*. In particular there are no constraints on the pathway searched at the root node, corresponding to the first call to *FindPath*, this fact is expressed by having empty *R_f _*and *R_n_*. The hyperpath found is *R*_1_, *R*_2_, *R*_4_, *R*_7_. For each reaction in the hyperpath, a new call to *FindPath *is done, this time with the constraints induced by the hyperpath solution of the parent node. Processes that do not return hyperpaths (P={∅}) are not followed by calls to children processes, while the processes that return one hyperpath P have unconstrained reactions $P\backslash R_{f}$ and are followed by as many children processes as unconstrained reactions. The children processes have as set of reactions that cannot belong to the returned pathway *R_n_*' the same as the father augmented by one reaction from the unconstrained set of the hyperpath P returned by the father process, while the set of reactions required to belong to the pathway *R_f _*' is given by the ones of the father augmented by all the reactions preceding the one added to *R_n _*to get *R_n_*'. Consider for instance the process giving as pathway $P=\left\{R_{1},R_{2},R_{5},R_{9}\right\}$ it contains two reactions not in *R_f _*: *R*_5 _and *R*_9 _so its two children processes have *R_n _*given by *R*_4 _∪ *R*_5 _and *R*_4 _∪ *R*_9 _and *R_f _*is given by the one of the father: {*R*_1_, *R*_2_} union once with {∅} and once with *R*_5_: the only unconstrained reaction preceding *R*_9 _in the pathway P.

First we describe informally the enumeration algorithm through the toy example hypergraph in Figure 2D and 3A, then we outline in Figure 3B a typical run for a more involved example: liquiritigenin (cf. Figure 1).

A minimal hyperpath $P_{1}$ connecting the node *v*_8 _to the source nodes *v*_1_, *v*_4 _on the hypergraph H of Figure 2D can be obtained by calling *Minimize *($P^{\prime }$, {∅}, {*v*_8_}, {*v*_1_, *v*_4_}) on the hypergraph $P^{\prime }$ obtained by $FindAll\left(H,\left\{v_{1},v_{4}\right\}\right)$. The hypergraph $P^{\prime }$ is represented in Figure 3A.

Once $P_{1}=\left\{R_{4},R_{3}\right\}$ has been obtained, the search space is divided into three parts:

• the hypergraphs which do not contain *R*_4_,

• the hypergraphs which do contain *R*_4 _and do not contain *R*_3_,

• the hypergraphs which do contain *R*_4 _and *R*_3_.

The first set does not contain hyperpaths connecting the target to the source: once the reaction *R*_4 _is removed, *v*_8 _is disconnected from the source. The second set contains a solution and thus has to be partitioned. The third set contains only one minimal pathway (the one consisting of hyperarcs *R*_3_, *R*_4 _highlighted in Figure 3A).

The minimal hyperpath in the second set is found by running *FindAll *on $H\backslash R_{3}$ and then *Minimize *with constraint *R_f _*= {*R*_4_}. The minimal hyperpath so obtained is the one only containing hyperarcs *R*_4_, *R*_7_. The set of the hypergraphs defined by (*R_f _*= {*R*_4_}, *R_n _*= {*R*_3_}) is partitioned in two parts defined by new sets of constraints. The way the partition is done is explained in detail in algorithm *FindPath *and gives two non overlapping sets:

• the hypergraphs which do contain *R*_4 _and do not contain *R*_3 _and *R*_7_.

• the hypergraphs which do contain *R*_4 _and *R*_7 _and do not contain *R*_3_.

The first of these sets does not contain hyperpaths going to *v*_8_: once *R*_3 _and *R*_7 _are removed, node *v*_8 _is disconnected from the source. The second one only contains the second and last minimal hyperpath: the one consisting of hyperarcs *R*_7_, *R*_4_. The algorithm here sketched is based on the fact that all minimal hyperpaths are found once the problem of finding a minimal hyperpath has been solved for each part of the partition.

#### Relaxed hyperpath minimization

The enumeration procedure is performed by the algorithm *FindPath*, which enumerates all the minimal pathways and does not output duplicate hyperpaths. Precisely *FindPath *(H, *R_f_*, *T*, *$S_{H}$*) returns a set of hyperpaths containing all the minimal hyperpaths in H connecting *T *to $S_{H}$ and containing all the reactions in *R_f_*. *FindPath *(H, {∅}, *T*, *$S_{H}$*) returns all the minimal hyperpaths from the source $S_{H}$ to the target *T *in H.

A schematic representation of how *FindPath *works for the enumeration of the pathways of liquiritigenin is given in Figure 3B where we represent each call with a box connected by an arrow to its parent process. For each call of *FindPath *either a new hyperpath is found and then *FindPath *is executed with new constraints, or there are no new hyperpaths and the branching process is stopped. The new constraints sets *R_f _*', *R_n_*' for a new call of *FindPath *are obtained by incrementing the sets *R_f_*, *R_n _*of the father process. Given an order for the hyperarcs of the hyperpath P found for the father process, the set *R_n_*' relative to the child process is constructed by incrementing *R_n _*by one element *r *belonging to P, the set *R_f _*' is constructed by incrementing *R_f _*by all the hyperarcs coming before *r*. For each element in P not belonging to *R_n _*a child process is called.

*FindPath *(H, {∅}, *T*, *$S_{H}$*) returns all the minimal hyperpaths from the source $S_{H}$ to the target *T *in H. In the context of metabolic engineering *FindPath *returns all the metabolic pathways for the production of the target compounds.

**Algorithm FindPath **(Enumerate all minimal hyperpaths from $S_{H}$ to the target set *T *with constrains *R_f _*on the hypergraph given by H)

input:

H, *R_f_*, *T*, *$S_{H}$*

1. ***F ***← FindAll(H,$S_{H}$)

2. P ← Minimize(∪*_k_F_k _*∪ *R_f_*, *R_f_*, *T*, *$S_{H}$*)

3. *En *← ∅

4. **if P≠∅**

5.    En←P

6.       $F\leftarrow \text{FindAll}\left(P,S_{H}\right)$

7.       **for all ***k ***in **|***F***|,..., 1}

8.          *r *= *F_k_*

9.          **if ***r ***not in ***R_f _*:

10.             $En\leftarrow \left\{En,\;\text{FindPath}\left(H\backslash r,R_{f},T,S_{H}\right)\right\}$

11.             *R_f _*← *R_f _*∪ *r*

12.          **end if**

13.       **end for**

14.    **end if**

output:

   *En*

The loop at line 7 of *FindPath *is done according to the order given by line 6 where the hyperarcs are ordered so that at least one of the head vertices of each hyperarc is a tail vertex of some previous reaction. Such an ordering is always possible since P is a hyperpath. As said above and illustrated in Figure 3B, F*indPath *is an algorithm that iteratively calls itself, see line 10. Note that even if *R_n _*is not explicitly defined in *FindPath*, it is constructed implicitly when at line 10 of *FindPath *is called on the smaller graph H\r.

Let us note that the output of the enumeration is not always composed of minimal hyperpaths. This is due to the fact that the algorithm *Minimize *while running in polynomial time can return a non-minimal hyperpath. An algorithm always returning minimal hyperpaths cannot be polynomial since the problem of finding a minimal hyperpath containing a set *R_f _*of hyperarcs is an NP-complete problem as showed in Appendix A.2. However, in many practical instances (for instance when hyperarcs only have one head node), the algorithm *Minimize *returns a minimal constrained hyperpath. As a matter of fact, for all the enumeration studies we have so far carried out, we observed that the output obtained by *Minimize *when called by the algorithm *FindPath *introduced above was always a minimal hyperpath. Nonetheless, a characterization of hard instances of the minimal constrained hyperpath problem is given in Appendix.

#### Supplements Enumeration

Provided a given metabolic network and a set of source compounds (e.g. a set of compounds in the growth media, a set of endogenous metabolites of a species) it may not be possible to link all the metabolites of the network to the source set. When a target compound is not accessible from the source set, one can consider the possibility of inserting into the metabolism of the organism some precursors so that the target becomes reachable. In practice such a task can be carried out through the enrichment of the growth media. More generally, the insertion of supplements can be used even when the target compound is reachable in order to access to new pathways for the production of the target.

Let a supplement for a target *T *be any compound $i\notin B\left(H,S_{H}\right)$ that is involved as reactant in at least one minimal hyperpath going from a superset of $S_{H}\cup i$ to the target *T*. Below we give the algorithm *FindSupp *finding the supplements for the production of a given target. In Figure 2D supplements are highlighted in red. Therefore, the process of finding supplements is use-ful as a general strategy in metabolic engineering in order to determine which metabolites might be part of the metabolism that produces a given target. **Algorithm FindSupp **(Find supplements for the production of the compounds in *T*, from the source $S_{H}$ of hypergraph H)

input:

H, *$S_{H}$*, *T *(list of compounds to produce)

1. *WishList *← *T*

2. *D *← {∅}

3. **while ***WishList \ D *≠ {∅}

4.    let *i *be an element of *WishList \ D*

5.    *D *← *D *∪ *i*

6.    *Aux *← {∅}

7.    **for all **reactions *r *with *i *∈ *Y *(*r*)

8.       $Aux\leftarrow Aux\cup \left(X\left(r\right)\backslash \left(S_{H}\cup D\right)\right)$

9.    **end for**

10.       *WishList *← *WishList *∪ *Aux*

11. **end while**

12. $F\leftarrow \text{FindAll}\left(H,S_{H}\right)$

13. $D\leftarrow D\backslash S_{H}\cup \left(\cup_{k}Y\left(F_{k}\right)\right)$

output:

D

#### Bootstraps

Bootstrap molecules relative to a source $S_{H}$ are the molecules that cannot be produced by a hyperpath with source $S_{H}$ unless they are already present in the media. An example of bootstrap nodes are nodes *v*_2_, *v*_3 _of Figure 2D. In this section we give an algorithm finding in polynomial time all the bootstraps of a hypergraph H with source vertices $S_{H}$. Bootstraps are special kind of supplement, if at any step of a pathway, a heterologous metabolite is needed as a substrate but has not yet been produced from the source set, then this metabolite is a bootstrap and must be added in the growth media for the reaction to take place, and for the pathway to be a valid pathway. The algorithm given below enables one to detect bootstraps prior enumerating pathways running the *FindPath *algorithm.

**Algorithm FindBootstraps **(Given a hypergraph H and a source $S_{H}$, returns the set *B *of bootstrap nodes)

input:

$H,S_{H}$

1. $F\leftarrow \text{FindAll}\left(H,S_{H}\right)$

2. $D\leftarrow S_{H}\cup \left(\cup_{k}Y\left(F_{k}\right)\right)$

3. $H^{\prime }\leftarrow \left\{\emptyset \right\}$

4. **for all ***r ***in H**

5.    *r*' ← (*X *(*r*) *\ D*, *Y *(*r*) *\ D*)

6.    **if ***Y *(*r*') ≠ {∅}:

7.       $H^{\prime }\leftarrow H^{\prime }\cup r^{\prime }$

8.    **end if**

9. **end for**

10. **while **exists *v ***in $\cup_{r\in H^{\prime }}Y\left(r\right)\backslash \cup_{r\in H^{\prime }}X\left(r\right)$**

11.    **for all ***r*' containing *v*:

12.       *r*' ← (*X*(*r*'), *Y*(*r*') *\ v*)

13.       **if **(*Y*(*r*') = {∅}) **or **(*v *∈ *X*(*r*')):

14.          $H^{\prime }\leftarrow H^{\prime }\backslash r^{\prime }$

15.       **end if**

16.    **end for**

17. **end while**

18. $B=\cup_{r\in H^{\prime }}Y\left(r^{\prime }\right)$

output:

   B

The *FindBootstraps *algorithm is linear in the number of vertices, hyperarcs and in the total coordination. Remark that the set $\cup_{r\in H^{\prime }}Y\left(r^{\prime }\right)$ obtained in line 18 is equal to $\cup_{r\in H^{\prime }}X\left(r^{\prime }\right)$. In fact the bootstrap vertices $b\;\left(H,S_{H}\right)$ constitute the largest set of vertices not reachable from the source and such that each element of the set belongs to the head of at least one reaction whose tail only contains vertices in $B\left(H,S_{H}\right)$ or in $b\;\left(H,S_{H}\right)$. Notice that the set of bootstrap vertices in a hypergraph H only depends on the source vertices and does not depend on the target.

## Results and Discussion

To evaluate the performance of our topological approach (e.g. algorithm FindPath), we have compared running times of this approach with the running times of the steady state approach presented in the Methods section. All our tests were run on a Mac Pro server 2 *× *2.66 Ghz Quad-Core Intel Xeon, 16 GB. All the algorithms of the topological approach were implemented in Python. For the steady state approach we used two software products, one computing elementary modes and the other extreme pathways. Elementary modes were computed by using the MATLAB interface to the Java implementation of efmtool Version 4.7.1 [7]. Extreme pathways were computed by using the Mac OSX version of the ExPA program [6]. The running time comparison test was performed for different random samples of the hypergraph H constructed from the KEGG database [27]. *E*. coli was chosen as source organism. The hypergraph H was composed of 6542 metabolites connected by 8392 reactions including 971 metabolites endogenous to *E*. coli and 5571 heterologous compounds. Each sampled hypergraph $H_{s}$ was built by randomly sampling a given fraction of the total reactions in the hypergraph H. Tests for each sampling fraction were repeated 10 times. In the case of elementary modes, we were able to run the test only up to 50% sampling of the full metabolic network, due to memory constraints in MATLAB. For extreme pathways, the test was run up to 85% due again to memory constraints as well. Prior to enumerating pathways with elementary modes, extreme pathways or the direct algorithm, bootstraps were identified by using the algorithm *FindBootstraps *defined in Methods and added to the growth media.

**Figure 4:**
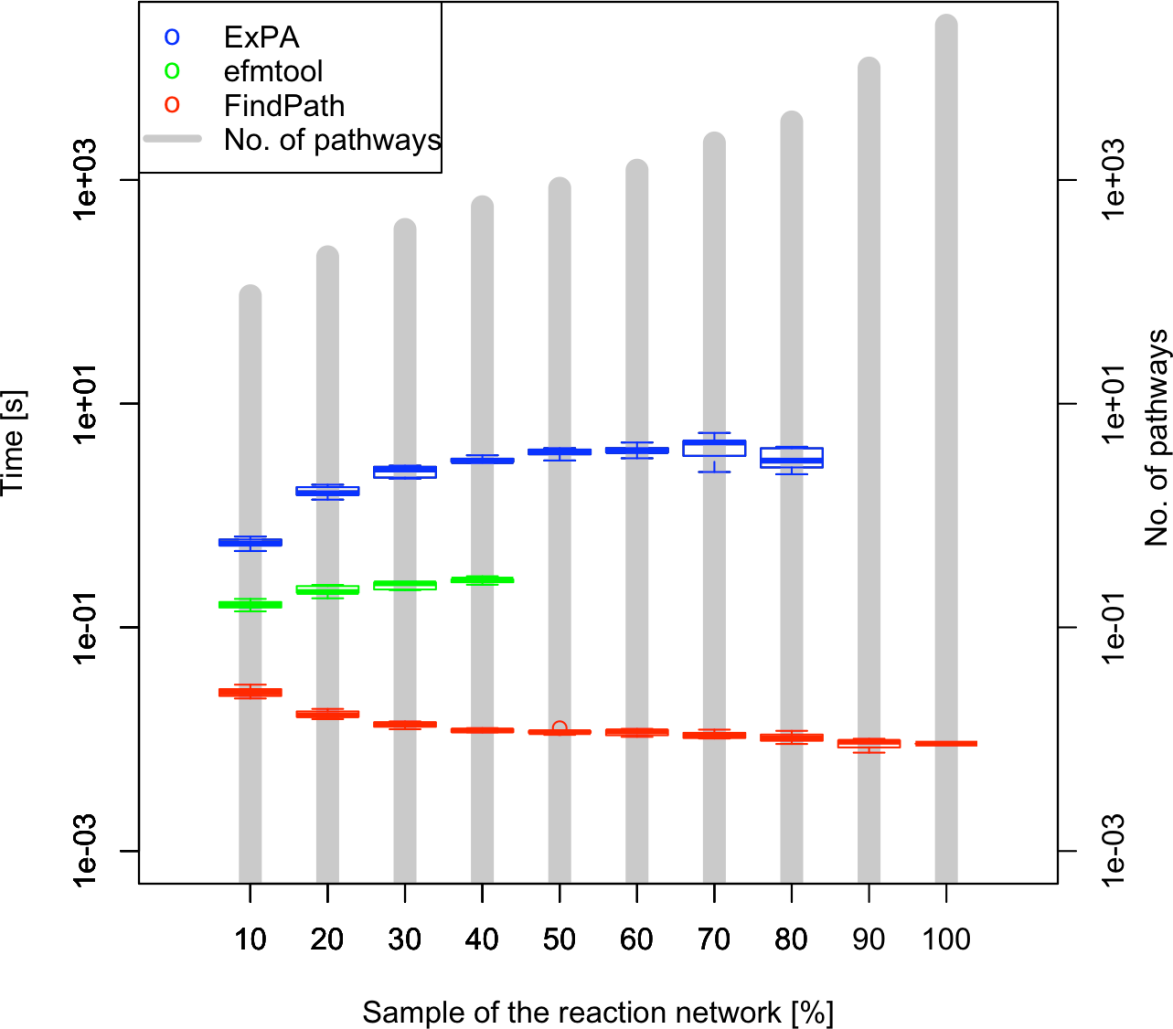
**Run times per output by using **FindPath**, elementary modes **(efmtool)**, and extreme pathways (**ExPA**)**. The average total number of pathways for each fraction of the network is shown in gray.

The observed trend is consistent in the three cases, as it is shown in Figure 4. For the network composed of all 6542 metabolites and 8392 reactions, there are 23564 pathways connecting 2338 out of 5571 heterologous metabolites to the source. This relative low number of pathways producing heterologous compounds is related to the fact that heterologous maps usually show a degree of hierarchy higher than the one observed in native metabolic networks such as central metabolism (a comparison is provided in supplementary Additional file 1, Figure S1). It is indeed not surprising to find the same number of extreme pathways and elementary modes as all reactions in our networks are irreversible. In the case of FindPath, the fact that the topological approach does not take stoichiometry into account might produce some inconsistent pathways. Checking that a given pathway is stoichiometrically balanced can be done using linear programming [28]. In the context of metabolic engineering (i.e. enumerating pathways between native metabolites and heterologous targets) we found only few inconsistent pathways (less than 1% of the 23564 enumerated pathways). These pathways are eliminated after enumeration by our ranking function as one of the criteria to rank pathways is to solve the steady state equations [1]. Once unbalanced pathways were removed from the list of enumerated pathways, we obtained the same pathways as through the calculation of elementary modes. These results are consistent with the given definitions of the algorithms. In a forthcoming paper, we plan to generalize them into a formal proof.

We also find that FindPath has the fastest execution time, the algorithm required less than 5 min to compute the full network. Elementary modes has an execution time approximately 10-fold slower than FindPath. The computation of extreme pathways, in turn, is the slowest one, being between 10^2 ^~ 10^3^-fold slower than FindPath. The execution times are on average of 0.0136 ± 0.0051 s per output with the FindPath algorithm, of 0.218 ± 0.042 s with elementary modes, and of 2.84 ± 1.24 s with extreme pathways. The observed execution times mean that for the compound with the highest number of pathways in our tests, the anti-diabetic drug acarbose containing 1513 pathways, enumerating the pathways with elementary modes takes more than 329.83 seconds, while it can be computed in 20.58 seconds with FindPath. We have measured running times per output and memory usage as function of input size and output size (see Table 1 and Additional file 2, Figure S2). In all cases we found linear growth *O*(*n*) for both input size and output size. In the case of running times, FindPath and efmtool have running times per output approximately constant in function of the size of the input stoichiometric matrix S of 3.1 × 10^-2 ^and 1.6 × 10^-1 ^seconds, respectively, being ExPA the less efficient code with a constant time of 4.2 × 10^*-*1 ^seconds and a linear growth of 1.6 × 10^*-*7 ^seconds per input. Similar values were obtained depending on the size of the output, although the scaling in this case for ExPA was of 5.7 × 10^*-*3 ^seconds per output. Regarding memory usage, ExPA and FindPath are less demanding, especially for output size, with linear growths of 3.9 and 54 kB per output, respectively, while efmtool required significantly more memory allocation, 3.3 × 10^4 ^kB per output.

**Table 1:** Performance comparisons

	Code	Run time per output [s]	Memory use [kB]
Size of input **S **[*n *× *m*]	FindPath	3.1 × 10^-2 ^- 1.1 × 10^-9^*x*	4.2 × 10^+1 ^+ 1.5 × 10^-2^*x*
	efmtool	1.6 × 10^-1 ^+ 6.7 × 10^-9^*x*	2.0 × 10^+5 ^+ 9.6 × 10^-2^*x*
	ExPA	4.2 × 10^-1 ^+ 1.6 × 10^-7^*x*	3.3 × 10^+3 ^+ 1.1 × 10^-5^*x*
Size of output [no. of pathways]	FindPath	3.2 × 10^-2 ^- 4.0 × 10^-5^*x*	-1.6 × 10^+1 ^+ 5.4 × 10^+1^*x*
	efmtool	1.5 × 10^-1 ^+ 2.3 × 10^-4^*x*	1.0 × 10^+5 ^+ 3.3 × 10^+4^*x*
	ExPA	2.5 × 10^-1 ^+ 5.7 × 10^-3^*x*	3.2 × 10^+3 ^+ 3.9 × 10^+0^*x*

Performance of the codes FindPath, efmtool and Expa in terms of run time per output and memory use in function of size of input stoichiometric matrix **S **and size of output (number of pathways).

To the best of our knowledge, the computational complexity of enumerating elementary modes on networks comprising irreversible reactions is up-to-date unknown [28]. In Appendix A.2 we prove that enumerating minimal pathway is an NP-complete problem (reduction to 3-SAT) but as examplified in Appendix B the hard instances are rather rare and obviously none of these hard instances were found in our running tests as all pathways returned by FindPath were indeed minimal pathways.

**Figure 5:**
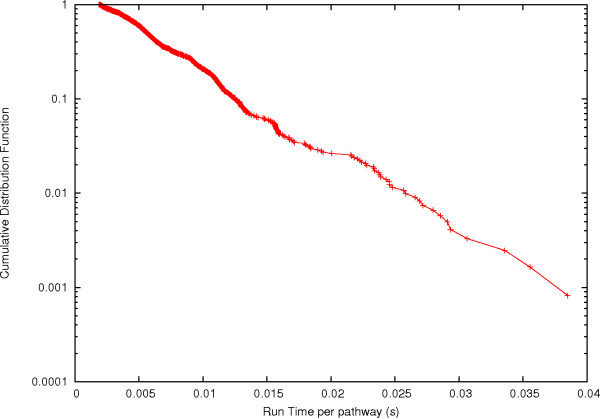
**Cumulative probability distribution function of the run time per pathway**. The probability that a randomly selected target requires a solution time *t *≥ *T *is represented and its asymptotic behavior for large *T *appears to be exponential.

To further probe the computational complexity of FindPath we computed distribution of the run time for each of the 2338 heterologous targets that are linked to *E*. coli. As shown in Figure 5, the distribution is exponentially distributed, the average run time is thus finite. Examples of pathway enumeration for heterologous compounds with therapeutical applications were provided in our previous methodology study about pathway ranking [1] for penicillin N and taxol (see also Additional file 4, Figure S5 and S6). These two examples contained a relative low level of combinatorial complexity, with a maximum number of 14 different pathways producing penicillin N. The combinatorial complexity of enumerating all putative biosynthetic routes producing a target compound, however, might become considerably higher than in these two previous examples (cf. Figure 2 in [1] where more than 10000 pathways can be found between tyrosine and chorismate). Further examples are those involved in the biosynthetic pathways for plant steroids leading to brassinolide, where 8 pathways can be used to produce campesterol, one of the initial precursors. The number of pathways grows as we proceed downstream going up to 40 for teasterone, and to 224 for typhasterol. Finally, the number of pathways for the end products, castasterone and brassinolide is of 328. This example illustrates how the complexity of pathways enumeration can grow with the number of intermediates involved in the synthesis of the final product. Since FindPath algorithm has been designed with the aim to enumerate pathways in metabolic engineering applications, it does not directly address other general metabolic network analysis problems like finding shortest paths between two compounds of endogenous pathways in central metabolisms. Indeed, FindPath does not enumerate pathways comprising compounds (bootstraps) that are consumed before being produced unless they were added as supplement in the growth medium (cf. in Additional file 3 an example of pathway enumeration with FindPath using bootstrap compounds).

Another aspect of pathway design is the definition of a cost function that estimates limiting effects on production efficiency by multiple factors associated with genetic and metabolic engineering of the pathway, such as gene heterogeneity, metabolite toxicity, or steady-state fluxes. In order to facilitate the designer in the selection of the best synthetic pathways to be implemented each hyperpath enumerated by our *FindPath *algorithm is ranked depending on this cost estimation. Once a cost function to minimize is introduced, the search for an optimal pathway can be formulated as a shortest path problem. The shortest path problem for weighted graphs consists of finding a path going from a given source vertex to a given target vertex while minimizing a cost function given by the sum of the costs of the arcs involved in the path. In order to define a shortest-hyperpath problem we need a definition of cost for hyperpaths in a weighted hypergraph (i.e. a hypergraph whose hyperarcs have associated a non-negative real number representing their cost). A natural generalization of the cost function for paths in graphs to hypergraphs is the sum of the costs of the hyperarcs contained in the hyperpath. If on the one hand this generalization seems to be natural, on the other hand the two problems have, however, not the same complexity. In fact the shortest-hyperpath problem with this cost function is known to be an NP-hard problem (see Appendix A.1).

The reason why the algorithms commonly used for graphs cannot be easily adapted for hypergraphs is that given the costs $W\left(P,a_{i}\right)$ for the hyperpaths $A_{i}\subset P$ leading to the vertices *a_i_*, tails of a hyperarc *e_j _*whose cost is *w_j_*, then the cost of the hyperpath containing all the hyperarcs in ∪*_i_A_i _*and *e_j _*is not equal to $w_{j}+\sum_{i}W\left(P,\;a_{i}\right)$ if the overlap of the hyperpaths *A_i _*is non trivial.

Provided an additive cost function as defined in [23], finding the shortest hyperpath can be done in polynomial time by an algorithm of complexity *O*(*m *· (*n *+ ln *m*)) given in [23], which finds the shortest hyperpath in *B*-hyperpaths. In fact, this algorithm does apply to minimal hyperpaths given in definition (4).

Generally speaking, the strategy we used in the enumeration algorithm (iterative partition of the space of feasible solutions) is often used to solve the problem of finding the first *k *solutions of a combinatorial problem, but it cannot be extended to the *k*-shortest problem on hypergraphs not even with an additive cost function (cf. definition in Appendix A.1), since when splitting the problem into a constrained part and a free one (by using the sets *R_n _*and *R_f _*) we get a problem that is known to be NP-hard (see the proof in [29] where an algorithm is given for the *k*-shortest hyperpath on hypergraphs whose hyperarcs have only one head vertex).

Therefore, the *k*-shortest path problem might be solvable efficiently if one is able to develop a specific cost function making the problem tractable. However such an artifact cost function may not be necessarily appropriate to practical problems, such as the production of heterologous targets using metabolic engineering. As an alternative we have proposed in this paper an enumeration algorithm, which systematically enumerates all pathways linking source compounds to target compounds. In all the cases that we have so far processed for metabolic networks, our algorithm runs in polynomial time per output, and therefore pathways can be ranked by the designer based on their own user-defined cost functions.

## Conclusions

In summary, the methods presented in this paper provide metabolic engineers with powerful tools that extend the toolbox for heterologous biosynthetic pathway design. Besides pathway enumeration of biosynthetic routes for a given target product, our methods have several other possible applications. For instance, they can be used in combination with gene deletion strategies in order to determine pathway manipulations leading to overproduction of the target compound. Another application is in biodegradation and bioremediation, where our algorithms would need to be slightly modified in order to reverse the pathway search so that it can identify degradation routes for a given compound, while the underlying structure of the algorithms remains still valid. Finally beyond metabolism, our algorithms could also be utilized in the context of chemical synthesis to enumerate all the possible routes linking a target molecule to a source set of starting reactants, enabling the search for the best routes in terms of production costs.

## Availability and requirements

A web server is available: http://bioretrosynth.issb.genopole.fr/tools/metahype See details in Additional file 4.

## Authors' contributions

JLF and PC designed the algorithms and the experiments. DF implemented the algorithms, and wrote the proof of the NP-completeness for the minimal constrained hyperpath problem. SBP wrote the example of pathway enumeration. PC implemented the web service prototype, performed the experiments and analyzed the results. All authors wrote the manuscript. All authors read and approved the final manuscript.

## Appendix A Reduction proofs for the shortest hyperpath problem and the minimal constrained hyperpath problem

### A.1 Shortest hyperpath Problem

In [30] a reduction of the shortest-hyperpath problem to Minimum Set Cover (MC) is given. We have to adapt the proof to our case for two reasons: the definition of directed hypergraph that was used is more restrictive (they only admit hyperarcs *e_i _*such that *|Y *(*e_i_*)| = 1) and the hyperpath was ill-defined. In fact the given definition by these authors does not permit to say if some hypergraphs (as the one in Figure 2C) are also hyperpaths or not. In other words their definition is ambiguous: does not permit to determine the nature of all the directed hypergraphs and thus can be completed in several ways.

Nonetheless the proof of NP-hardness they gave is valid for our more general hypergraphs and minimal hyperpaths because the set of directed hypergraphs employed in the reduction proof in [S1] is a sub-ensemble of the directed hypergraphs we defined above in the Definitions section and all the hypergraphs employed for the reduction are well defined as hyperpaths, independently of the way the incomplete definition they gave is completed. Since our definition is a way to complete the definition in [S1], then the two definitions agree on the set of hypergraphs employed for the reduction.

From these facts follows that the shortest hyperpath problem is an NP-hard problem. And, in particular, if the weights on hyperarc are non-negative, since hyperpaths always contain at least one minimal hyperpath, the shortest minimal hyperpath problem is NP-hard too.

#### Additive cost functions

The reason why the algorithms commonly used for the shortest path problem on graphs cannot be easily adapted for hypergraphs is that given the costs $W\left(P,a_{i}\right)$ for the hyperpaths $A_{i}\subset P$ leading to the vertices *a_i_*, tails of a hyperarc *e_j _*whose cost is *w_j_*, then the cost of the hyperpath containing all the hyperarcs in $\cup_{i}A_{i}$ and *e_j _*is not equal to $w_{j}+\sum_{i}W\left(P,\;a_{i}\right)$ if the overlap of the hyperpaths $A_{i}$ is non trivial.

In order to define a shortest path problem that can be solved polynomially by a variant of Dijkstra algorithm, the additive cost functions have been introduced in [23] for the *B*-hyperpaths. We adapt below the notion of "additive" cost function for hyperpaths. A cost function W(P,x) returning the cost for reaching the vertex *x *with the hyperpath P starting from a source *S *whose elements *s *∈ *S *have *W*(*s*):= 0 is additive if *W *(*x*) is the minimum over all the arcs $e_{x}\in P$ whose head contains *x *of *e_x _*+ *f*(*W_i_*) where $W_{i}:=W\left(P,i\right)$ are the costs for reaching the tail vertices *i *of *e_x _*in P, *f *is an increasing monotone function of its argument and *f*(*W_i_*) *≥ W_i _*∀*i*. Remark that the cost of a hyperpath determined with an additive cost function in general is not given by the sum of the costs of the hyperarcs.

### A.2 Minimal Constrained Hyperpath Problem

Consider a 3-SAT instance concerning *n *variables *σ_i _*and *m *clauses *X_j _*consisting of the problem of deciding if there exists an assignment of Boolean values to the *σ_i _*such that all the clauses are satisfied. For each boolean variable *σ_i _*contained in at least one clause introduce one hyperarc *ε_i _*with the head of each *ε_i _*having two vertices *Y*(*ε_i_*) = {*v*_*i*+_, *v*_*i*-_}. For each clause *X_j _*consider a vertex *ν_j _*and seven hyperarcs (each one corresponding to boolean assignment of the three variables satisfying the clause *X_j_*). A boolean assignment is a triple *a*_1_, *a*_2_, *a*_3 _of boolean values. Let these hyperarcs be *μ*_*j*1_,..., *μ*_*j*7 _and let the head *Y*(*μ_jk_*) of a hyperarc *μ_jk _*corresponding to the combination *a*_1_, *a*_2_, *a*_3 _of the boolean variables *σ*_*j*1_, *σ*_*j*2_, *σ*_*j*3 _be *Y*(*μ_jk_*) = {*v*_*j*1*a*1_, *v*_*j*2*a*2_, *v*_*j*3*a*3_, *ν_j_*}. Now let the tails of each hyperarc introduced be connected to the source nodes. And let consider a node *T *being the product of the reaction *R *having as substrates the vertices *ν_j _*and the heads {*v*_*i*+_, *v*_*i*-_} of the hyperarcs *ε_i_*.

Given the hypergraph described above (whose size is linear in the size of the underlying 3-SAT problem) consider the minimal constrained hyperpath problem where all the hyperarcs *ε_i _*are mandatory, and the target is *T*.

A solution of this problem gives in linear time a solution for the underlying SAT problem, which makes the problem of minimal constrained hyperpath an NP-complete problem. In fact, given a minimal hyperpath *M*, solution of this problem, for each *i *consider $v_{i}^{*}$ the only one of the two head vertices {*v*_*i*+_, *v*_*i*-_} belonging to the head of one or more of the *μ *arc in M (only one of the two vertices can belong to the head of a *μ *hyperarc in M because otherwise the hyperarc *ε_i _*would be superfluous). The boolean assignments $\sigma_{i}=v_{i}^{*}$ are a solution of the 3-SAT problem and, inversely if a solution of the 3-SAT problem exists then a minimal pathway satisfying the constraints exists and is the one obtained using only one of the hyperarcs for each *X_j _*among the ones whose head only contains *ν_j _*and vertices $v_{i}^{*}$.

In Additional file 5, Figure S8 for simplicity we consider the reduction of a single-clause satisfaction problem to finding if a minimal hyperpath satisfying the constraints exists. There exist seven minimal hyperpaths connecting the target vertices to the source and satisfying the constraint that *ε*_1_, *ε*_2_, *ε*_3 _are parts of the hyperpath. Each solution corresponds to a valid boolean assignment of the variables *σ*_1_, *σ*_2_, *σ*_3_.

## Appendix B Hard instances of minimal constrained hyperpath problem

On many hypergraphs the algorithm enumerating the pathways only returns minimal hyperpaths, this is the case for the metabolic networks that we analyzed in the main sections of this paper. In this section we give a characterization of the hypergraphs where the algorithm *Minimize *solves the minimal constrained hyperpath problem, characterizing these instances helps to individuate which hypergraphs are expected to give an output only containing minimal hyperpaths.

Let *Y*(*R_f_*) be the set of all the metabolites produced by reactions in *R_f_*, the mandatory reactions: $Y\left(R_{f}\right):=\cup_{r\in R_{f}}Y\left(r\right)$. Given a hypergraph H and the sets *R_f_*, *R_n _*we say that the well-separation condition holds if for every reaction $r\in H\backslash \left(R_{f}\cup R_{n}\right)$ the set *Y*(*r*) of products of *r *either is a subset of *Y*(*R_f_*) or does not contain elements of *Y*(*R_f_*). If the well-separation condition holds for a hypergraph H with constrained reactions *R_f_*, the algorithm *Minimize *returns a minimal hyperpath solving the minimal constrained hyperpath problem if a solution exists.

The well-separation condition holds for every choice of *R_f _*in a hypergraph whose reactions have one only product, as the hypergraphs defined in [29]. If on the one hand, this condition can appear too constraining, on the other hand it can be generalized to larger sets of hypergraphs. For instance, the algorithm *Minimize *returns a minimal hyperpath solving the minimal constrained hyperpath problem even if the well-separation condition holds on the pruned graphs obtained by keeping from the original hypergraph only the reactions belonging at least to one hyperpath linking the target to the source and only the nodes being tail of such reactions.

Examples of hard instances can be found among the ones used for the proof of NP-completeness. In general, hard instances H of the enumeration problem have to violate the condition of well-separation for same choice of $R_{f}\subset H$, in order that the corresponding minimal constrained hyperpath problem becomes hard. This happens if several compounds are products of more than one reaction producing more than one compound.

This is the case for nested networks as the one in Additional file 6, Figure S9. While the given network is small enough to be solvable by hand, it contains nevertheless the principal ingredients of complexity that would asymptotically make harder the problem as the size of the instances grows.

Finding one minimal hyperpaths leading to the production of *v*_8 _is a simple problem, but finding new ones gets more and more involved. This is a consequence of the fact that the nodes *v*_1_, *v*_2_, *v*_3_, *v*_4_, *v*_5 _can be produced by different choices of the reactions *R*_1_, *R*_2_, *R*_3_, *R*_4_, *R*_5 _and each of these reaction has more of one product susceptible to participate to the production of the target.

## Supplementary Material

Additional file 1 **Figure S1**. Distribution of graph hierarchies (Butts, C *J Stat Soft*, 24:1-50, 2008) in heterologous metabolic networks (0.169 ± 0.11) in comparison with the graph hierarchy of central (0.032), nucleotide (0.027), lipid (0.051), and amino acid (0.030) metabolic networks in *E. coli*. Click here for file

Additional file 2 **Figure S2**. Performance comparisons for FindPath, ExPA and efmtools for run time per output and memory use in function of size of input and size of output. Click here for file

Additional file 3 **An example of pathway enumeration with bootstraps**. An example of pathways enumeration with Findpath using bootstraps. Click here for file

Additional file 4 **Availability and requirements**. Description of the available MetaHype web server for running the algorithms for KEGG compounds. Click here for file

Additional file 5 **Figure S8**. Minimal constrained hyperpath problem reduction of a 3-SAT formula. Click here for file

Additional file 6 **Figure S9**. Instance of minimal constrained hyperpath problem. Click here for file
