# Supplementary material for: Enumerating metabolic pathways for the production of heterologous target chemicals in chassis organisms
Source: BMC Syst Biol. 2012 Feb 6;6:10. doi: 10.1186/1752-0509-6-10 (PMC3311073; doi:10.1186/1752-0509-6-10)
Supplement: Additional file 1 — Figure S1. Distribution of graph hierarchies (Butts, C J Stat Soft, 24:1-50, 2008) in heterologous metabolic networks (0.169 ± 0.11) in comparison with the graph hierarchy of central (0.032), nucleotide (0.027), lipid (0.051), and amino acid (0.030) metabolic networks in E. coli. [file 1752-0509-6-10-S1.PDF]

Additional File 1

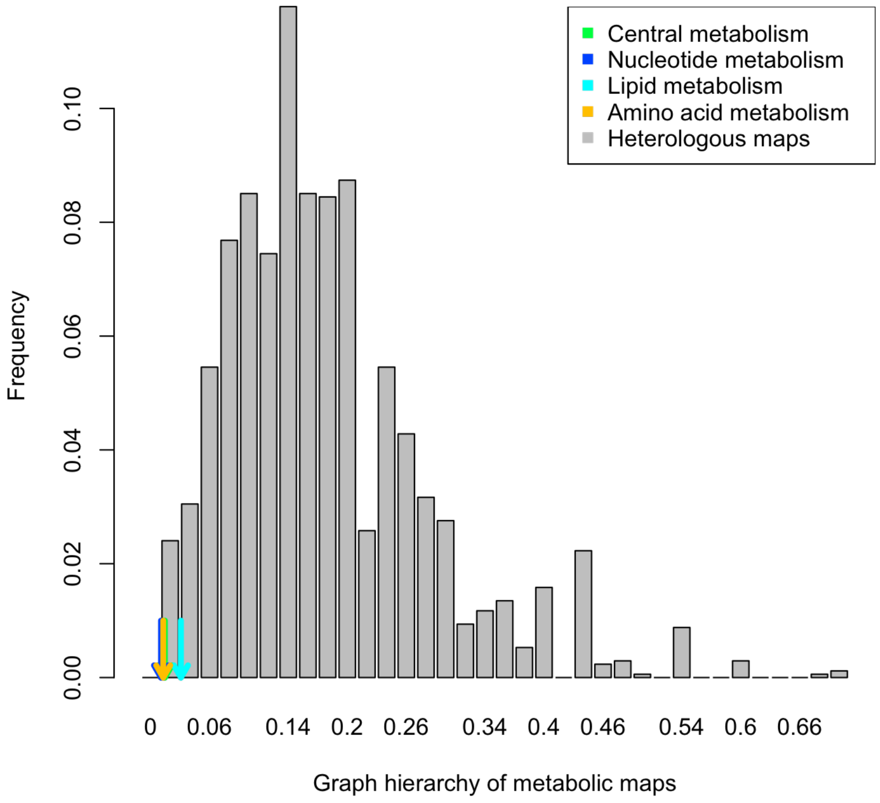

Figure S1: Distribution of graph hierarchies (Butts, C *J Stat Soft*, 24:1-50, 2008) in heterologous metabolic networks (0.169 ± 0.11) in comparison with the graph hierarchy of central (0.032), nucleotide (0.027), lipid (0.051), and amino acid (0.030) metabolic networks in *E. coli*.
