# Supplementary material for: Enumerating metabolic pathways for the production of heterologous target chemicals in chassis organisms
Source: BMC Syst Biol. 2012 Feb 6;6:10. doi: 10.1186/1752-0509-6-10 (PMC3311073; doi:10.1186/1752-0509-6-10)
Supplement: Additional file 2 — Figure S2. Performance comparisons for FindPath, ExPA and efmtools for run time per output and memory use in function of size of input and size of output. [file 1752-0509-6-10-S2.PDF]

Additional File 2

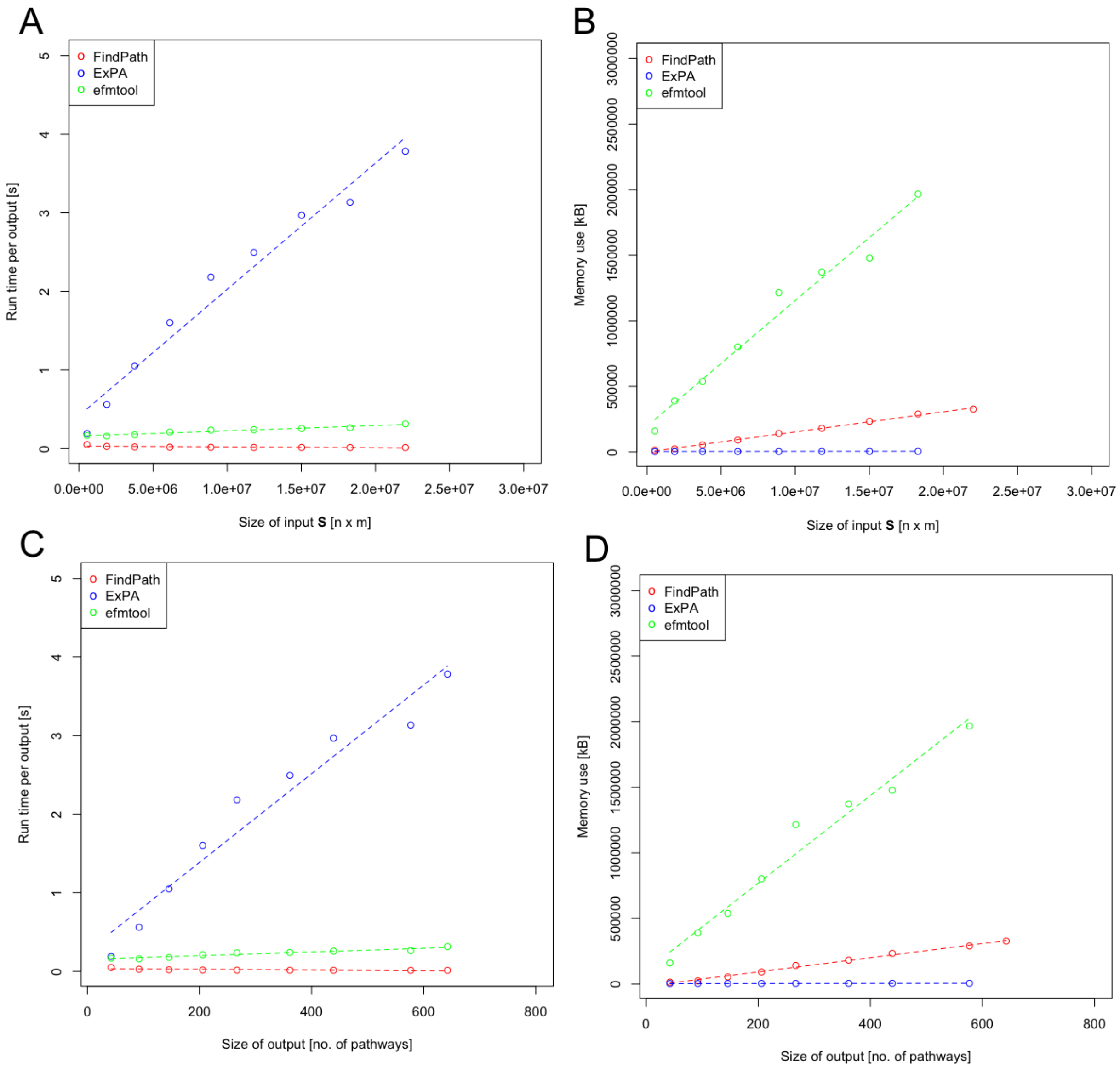

Figure S2: Performance comparisons for `FindPath`, `ExPA` and `efmtools` for run time per output and memory use in function of size of input and size of output.
