# Supplementary material for: Enumerating metabolic pathways for the production of heterologous target chemicals in chassis organisms
Source: BMC Syst Biol. 2012 Feb 6;6:10. doi: 10.1186/1752-0509-6-10 (PMC3311073; doi:10.1186/1752-0509-6-10)
Supplement: Additional file 3 — An example of pathway enumeration with bootstraps. An example of pathways enumeration with Findpath using bootstraps. [file 1752-0509-6-10-S3.PDF]

Additional File 3 - An example of pathway enumeration with FindPath using bootstraps

In the FindPath approach, we perform pathway enumeration in the context of metabolic engineering by searching for all possible heterologous pathways linking target compounds to the endogenous metabolites in a given chassis organism. In this example, we enumerate pathways from acetyl-CoA (AcCoA) to glucose. For this purpose we have defined a hypothetical chassis organism (*HS_org*), since most metabolites involved in TCA cycle, glyoxlyate shunt and gluconeogenesis pathways are usually endogenous. For this study, the designed system HS_org consists of reactions from the metabolite network (Figure S3) as described in Figueiredo et al., 2009 [1]. We considered AcCoA and currency metabolites *viz.* H_2O, ATP, NAD^+, NADH, NADPH, $NADP^+$, ADP, Orthophosphate, CoA, CO_2, NH_3, FAD, GDP, GTP, H^+, $HCO3^-$, $FADH_2$ as endogenous compounds. The endogenous compounds are "source" compounds and glucose is the "target". Using this system, we enumerated pathways that convert AcCoA to glucose by adding various metabolites (bootstraps) to the list of endogenous compounds.

The pathway enumeration performed using the list of endogenous compounds, as defined before, could not find paths that convert AcCoA to glucose. This is expected as the first enzymatic reaction in the TCA cycle, which consumes AcCoA requires oxaloacetate to be present in the chassis organism (i.e. in the list of endogenous compounds). Moreover, metabolites involved in TCA, glyoxylate shunt and gluconeogenesis are predicted as bootstrap molecules. Hence, we modified the list of endogenous compounds by adding to it various bootstrap metabolites. We used the modified lists of endogenous compounds to enumerate pathways in order to identify paths that can convert AcCoA to glucose involving enzymes from TCA, glyoxylate shunt and gluconeogenesis. The result of this study is summarized in Table S1.

Table S1: Summary of enumerated pathway for HS_org with endogenous metabolites and various bootstrap compounds added to the system

**Bootstrap compound**	**Number of enumerated pathways to produce Glucose**	**Glyoxylate shunt**	**TCA**	**Gluconeogenesis**
Pyruvate	2	-	-	+
Oxaloacetate / (Oxaloacetate + Pyruvate)	1	-	-	+
Malate / 2-oxoglutarate	2	-	+	+
Citrate / Isocitrate	6	+	+	+
Glycoxylate	2	+	+	+

The symbols (-) and (+) indicate absence and presence of reactions from the mentioned pathways respectively found in at least one of the enumerated pathways.

The list of bootstrap compounds consist of Pyruvate, 2-Oxoglutarate, Oxaloacetate, Citrate, Isocitrate, (S)-Malate, Succinate, Glyoxylate, Phosphoenolpyruvate, Succinyl-CoA, Glycerone, Alanine, Aspartate, Glutamate, D-Glyceraldehyde, Fumarate, beta-D-Fructose-1, 6-bisphosphate. We enumerated pathways with one or combination of bootstrap compounds added to our designed chassis organism. We present the enumerated pathways for representative bootstrap compounds. With oxaloacetate and/or pyruvate as bootstrap compounds for enumeration of pathways, we could not find paths, which converts AcCoA to glucose (Table S1). As is evident from the reaction network (Figure S3), oxaloacetate can be converted to glucose involving gluconeogenesis without AcCoA. This is expected given the fact that the present algorithm produces minimal hyperpaths according to Definition 4 in the manuscript. Similarly, with malate or 2-oxoglutarate as bootstrap compounds, we have 2 enumerated pathways that involve gluconeogenesis and reactions from TCA cycle (Table S1).

Interestingly, when we considered citrate or isocitrate or glycoxylate as bootstrap compounds with the chassis organism, we identified at least one pathway that consumes AcCoA to produce glucose involving reactions from glyoxylate shunt, TCA cycle and gluconeogenesis (Table S1). With citrate/isocitrate as bootstrap, we predicted 6 pathways that produce glucose that further is classified into two sets A_1 and A_2 based on involvement of AcCoA in the pathway. The set A_1 consists of 2 paths, which consumes AcCoA in the production of glucose and involve reactions from glyoxylate shunt, TCA cycle and gluconeogenesis (Figure S3). As described in Figuereido et al, 2009, these two pathways differ in the use of malate enzyme ME1 and pyruvate carboxylase versus malate dehydrogenase to produce oxaloacetate. The set A_2 consists of 4 pathways, which involve reactions from TCA cycle with gluconeogenesis (Figure S3) and does not consume AcCoA. Furthermore, we compared these with predicted pathways in Figueiredo et al., 2009 (Figure S3). As shown in figure S3, the main difference between the elementary modes found by Figueiredo et al., and FindPath predicted paths (set A_1) is that latter does not require succinate to be present in the system to replenish oxaloacetate to continue the TCA cycle, as is required in elementary modes. Moreover, since we provide citrate as bootstrap to the system, AcCoA and oxoaloacetate do not need to be consumed in order to synthetize citrate. The pathway with glycoxylate as bootstrap is part of pathway enumerated using citrate/isocitrate.

[1]. de **Figueiredo** LF, Schuster S, Kaleta C, Fell DA. Can sugars be produced from fatty acids? A test case for pathway analysis tools. ***Bioinformatics***. 2009 25:152-158.

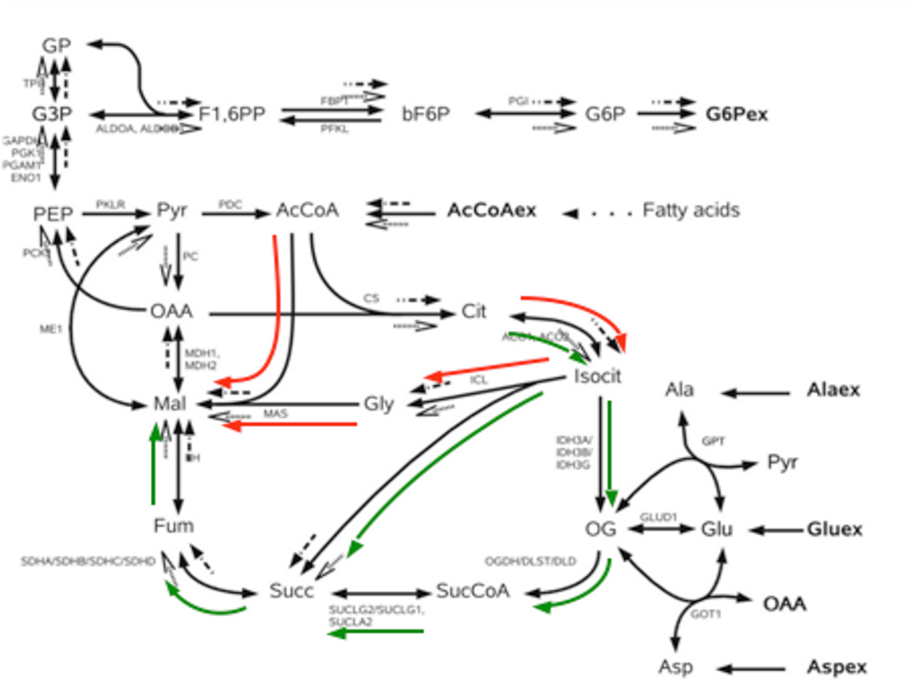

Figure S3: Comparison of elementary modes from Figueiredo et al., 2009 and FindPath predited pathways using Citrate/Isocitrate as bootstraps. The main difference in pathways of set A_1 and A_2 are colored in red and green respectively. The rest of paths is similar for set A_1 and A_2. The empty and full dashed arrows are elementary modes from Figueiredo et al., 2009. Abbreviations used are Pyr (Pyruvate), OG (2-Oxoglutarate), OAA (Oxaloacetate), Cit (Citrate), Isocit (Isocitrate), Mal ((S)-Malate), Succ (Succinate), Gly (Glyoxylate), PEP (Phosphoenolpyruvate), SucCoA (Succinyl-CoA), GP (Glycerone), G3P (D-Glyceraldehyde), Fum (Fumarate), F1,6PP (b-D-Fructose-1, 6-bisphosphate), Glu (Gluamate), Ala (L-Alanine), Asp (Aspartate), G6P (b Glucose 6-phosphate), 6F6P (b-D-Fructose-6-bisphosphate).
