# Supplementary material for: Enumerating metabolic pathways for the production of heterologous target chemicals in chassis organisms
Source: BMC Syst Biol. 2012 Feb 6;6:10. doi: 10.1186/1752-0509-6-10 (PMC3311073; doi:10.1186/1752-0509-6-10)
Supplement: Additional file 4 — Availability and requirements. Description of the available MetaHype web server for running the algorithms for KEGG compounds. [file 1752-0509-6-10-S4.PDF]

Additional File 4 - Availability and requirements

The algorithms can be executed for metabolic pathways for KEGG compounds through the MetaHype webserver at the following link:

http://bioretrosynth.issb.genopole.fr/tools/metahype

This webpage runs the software that performs the enumeration of all pathways for target compounds in KEGG. More precisely, the desired KEGG compound is submitted in order to get the map of all pathways connecting the compound to metabolites endogenous to *E.* coli. Optionally, the full map containing the supplements (see Methods) can also be requested.

Once the user submits the query, the output page contains (see also Figures S4-S7):

- **Map of reachable pathways**: An overview of all the pathways merged in a unique map is given as output. By clicking on this picture a new tab is opened on the browser containing a clickable larger picture. The nodes of the graph here represent either molecules or reactions and are linked to the corresponding web pages in KEGG.
- **Full map with supplements:** A clickable map is given where supplements are shown in yellow. This map is only plotted if the option Supplements map was selected in the main page.
- **Pathway list:** A summary table represents in a compact fashion the metabolic pathways leading to the target. Each row contains one pathway, the map of each single pathway can be accessed by clicking on the "id", and the rest of the row contains all reactions involved in the pathway, which are linked to the KEGG database. Finally, columns correspond to intermediate compounds in the pathway.
- **Export graph to Cytoscape:** a link to a file containing the graph in Cytoscape exchange SIF format is provided.

The MetaHype webserver is part of the retrosynthetic pathway design package. A beta version of that package can be accessed at at the bioretrosynth webserver.

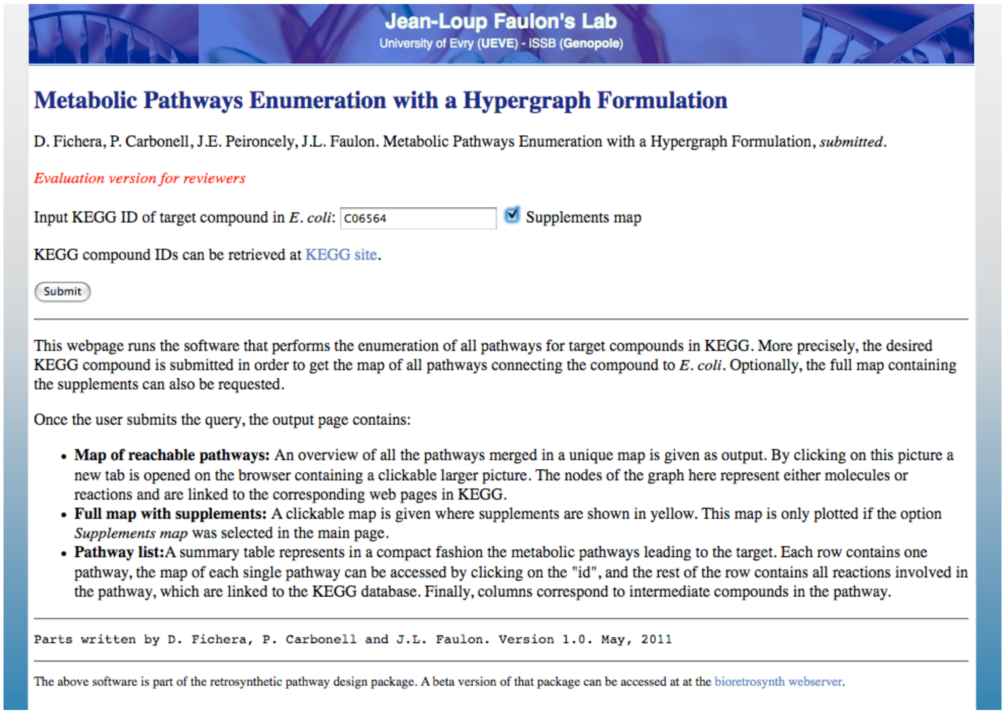

Figure S4: Access page.

Pathway enumeration for C06564

Map of reachable pathways

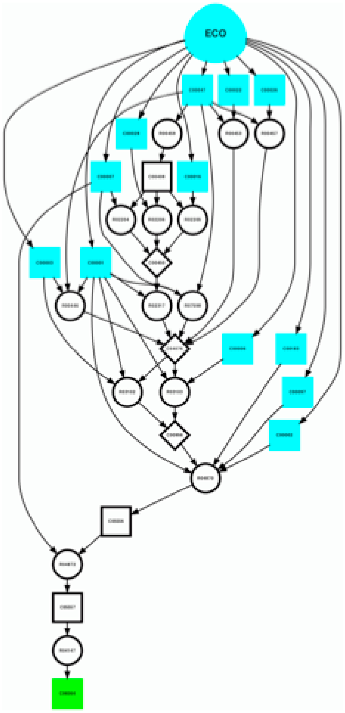

Full map with supplements

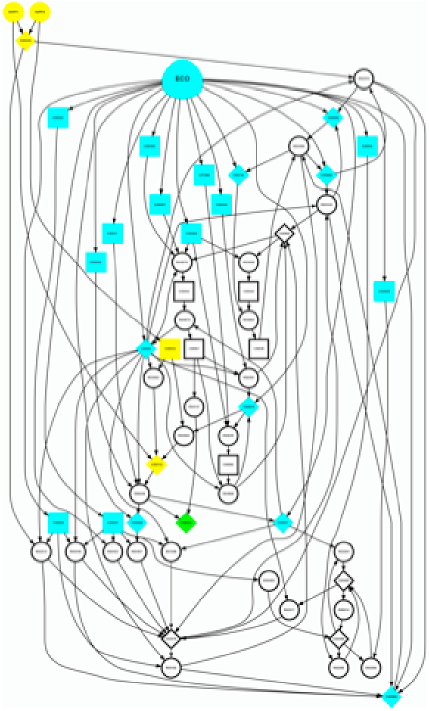

Pathways list

id	C00408	C00450	C00956	C04076	C05556	C05557	C06564
1	--	--	R03102	R00446	R04870	R04872	R04147
2	--	--	R03103	R00453	R04870	R04872	R04147
3	--	--	R03103	R00457	R04870	R04872	R04147
4	R00459	R02206	R03103	R02317	R04870	R04872	R04147
5	--	--	R03102	R00457	R04870	R04872	R04147
6	R00459	R02205	R03102	R02317	R04870	R04872	R04147
7	--	--	R03102	R07598	R04870	R04872	R04147
8	R00459	R02204	R03102	R02317	R04870	R04872	R04147
9	R00459	R02206	R03102	R02317	R04870	R04872	R04147
10	--	--	R03102	R00453	R04870	R04872	R04147
11	--	--	R03103	R07598	R04870	R04872	R04147
12	R00459	R02205	R03103	R02317	R04870	R04872	R04147
13	R00459	R02204	R03103	R02317	R04870	R04872	R04147
14	--	--	R03103	R00446	R04870	R04872	R04147

List limited to 100 pathways

Export graph to Cytoscape: SIF format

Figure S5: Main output page for the example compound "Penicillin N". A graphical representation of the map containing all pathways at once is shown. Single pathways (see Figure S3) can be accessed through the link in the summary table. A link to the Cytoscape export SIF format for the graph is also provided.

Pathway for C06564

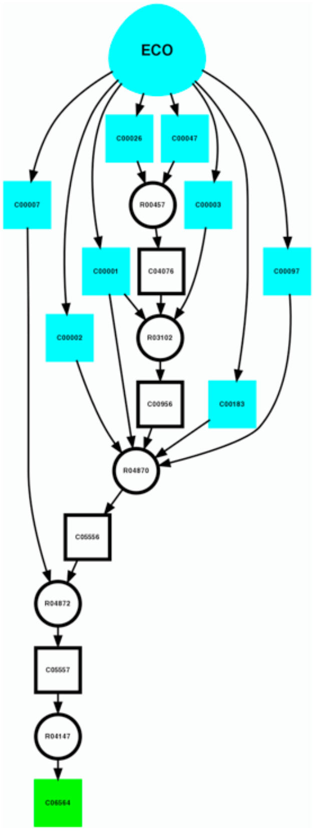

id	C00408	C00450	C00956	C04076	C05556	C05557	C06564
1	--	--	R03102	R00457	R04870	R04872	R04147

Export graph to Cytoscape: SIF format

Figure S6: Single Pathway. Single pathways can be accessed from the main output page (see Figure S2).

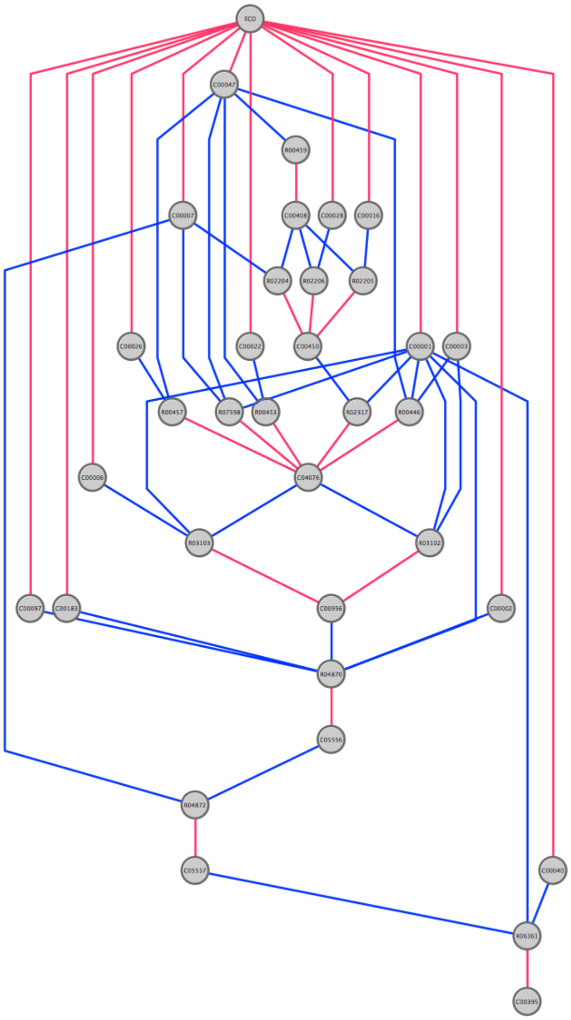

Figure S7: Cytoscape representation of the hypergraph using the SIF export file feature.
