# Supplementary material for: Enumerating metabolic pathways for the production of heterologous target chemicals in chassis organisms
Source: BMC Syst Biol. 2012 Feb 6;6:10. doi: 10.1186/1752-0509-6-10 (PMC3311073; doi:10.1186/1752-0509-6-10)
Supplement: Additional file 5 — Figure S8. Minimal constrained hyperpath problem reduction of a 3-SAT formula. [file 1752-0509-6-10-S5.PDF]

Additional File 5

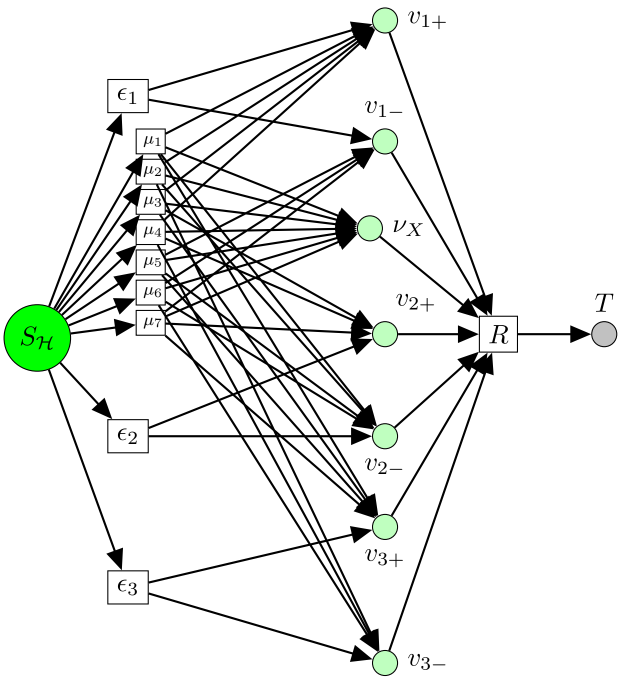

Figure S8: Minimal constrained hyperpath problem reduction of a 3-SAT formula. Satisfaction of formula $X = \sigma_1 \vee \neg\sigma_2 \vee \sigma_3$ is reduced to a minimal constrained hyperpath problem with hyperarcs $\{\epsilon_1, \epsilon_2, \epsilon_3\}$ constrained to be part of the minimal hyperpath. The target vertex is product of a reaction having as substrates two vertices for each boolean variable of the 3-SAT problem, and one vertex ν_X for each clause in X, in our example case only one.
