# Supplementary material for: Enumerating metabolic pathways for the production of heterologous target chemicals in chassis organisms
Source: BMC Syst Biol. 2012 Feb 6;6:10. doi: 10.1186/1752-0509-6-10 (PMC3311073; doi:10.1186/1752-0509-6-10)
Supplement: Additional file 6 — Figure S9. Instance of minimal constrained hyperpath problem. [file 1752-0509-6-10-S6.PDF]

Additional File 6

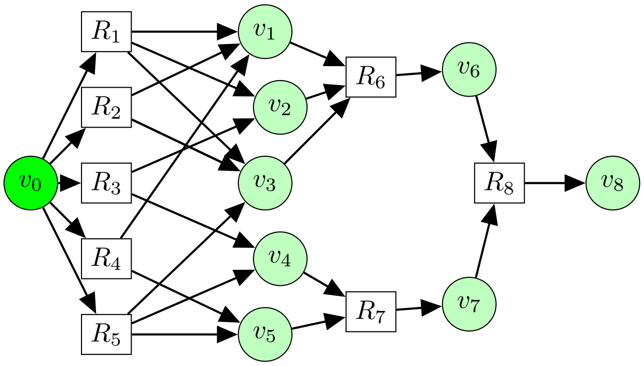

Figure S9: Instance of minimal constrained hyperpath problem. There are 5 minimal hyperpaths connecting the source v_0 to the target v_8.
